# Anaerobic *Faecalicatena* spp. degrade sulfoquinovose via a bifurcated 6-deoxy-6-sulfofructose transketolase/transaldolase pathway to both C_2_- and C_3_-sulfonate intermediates

**DOI:** 10.3389/fmicb.2024.1491101

**Published:** 2024-12-05

**Authors:** Sabrina Borusak, Karin Denger, Till Dorendorf, Corentin Fournier, Harry Lerner, Olga Mayans, Dieter Spiteller, David Schleheck

**Affiliations:** ^1^Department of Biology, University of Konstanz, Konstanz, Germany; ^2^The Konstanz Research School Chemical Biology, University of Konstanz, Konstanz, Germany; ^3^Department of Biology, Limnological Institute, University of Konstanz, Konstanz, Germany

**Keywords:** anaerobic microbial metabolism, carbon and sulfur cycle, isethionate, 3-sulfolactaldehyde, 6-deoxy-6-sulfofructose transaldolase, transketolase

## Abstract

Plant-produced sulfoquinovose (SQ, 6-deoxy-6-sulfoglucose) is one of the most abundant sulfur-containing compounds in nature and its bacterial degradation plays an important role in the biogeochemical sulfur and carbon cycles and in all habitats where SQ is produced and degraded, particularly in gut microbiomes. Here, we report the enrichment and characterization of a strictly anaerobic SQ-degrading bacterial consortium that produces the C_2_-sulfonate isethionate (ISE) as the major product but also the C_3_-sulfonate 2,3-dihydroxypropanesulfonate (DHPS), with concomitant production of acetate and hydrogen (H_2_). In the second step, the ISE was degraded completely to hydrogen sulfide (H_2_S) when an additional electron donor (external H_2_) was supplied to the consortium. Through growth experiments, analytical chemistry, genomics, proteomics, and transcriptomics, we found evidence for a combination of the 6-deoxy-6-sulfofructose (SF) transketolase (sulfo-TK) and SF transaldolase (sulfo-TAL) pathways in a SQ-degrading *Faecalicatena*-phylotype (family Lachnospiraceae) of the consortium, and for the ISE-desulfonating glycyl-radical enzyme pathway, as described for *Bilophila wadsworthia,* in an *Anaerospora*-phylotype (Sporomusaceae). Furthermore, using total proteomics, a new gene cluster for a bifurcated SQ pathway was also detected in *Faecalicatena* sp. DSM22707, which grew with SQ in pure culture, producing mainly ISE, but also 3-sulfolacate (SL) 3-sulfolacaldehyde (SLA), acetate, butyrate, succinate, and formate, but not H_2_. We then reproduced the growth of the consortium with SQ in a defined co-culture model consisting of *Faecalicatena* sp. DSM22707 and *Bilophila wadsworthia* 3.1.6. Our findings provide the first description of an additional sulfoglycolytic, bifurcated SQ pathway. Furthermore, we expand on the knowledge of sulfidogenic SQ degradation by strictly anaerobic co-cultures, comprising SQ-fermenting bacteria and cross-feeding of the sulfonate intermediate to H_2_S-producing organisms, a process in gut microbiomes that is relevant for human health and disease.

## 1 Introduction

The natural organosulfonate sulfoquinovose (SQ; 6-deoxy-6-sulfoglucose) constitutes the polar head group of sulfolipids (sulfoquinovosyl diacylglycerols, SQDGs), which are produced by most photosynthetic organisms (Benning, 1998; Douce et al., 1973; Sohlenkamp and Geiger, 2016) as part of the light-harvesting complex for oxygenic and anoxygenic photosynthesis (e.g., Sato et al., 1995; Martin et al., 2024). SQ can also be found as part of glycoproteins in the surface layer of archaea, such as *Sulfolobus acidocaldarius* (Meyer et al., 2011; Zähringer et al., 2000). SQ is considered to be one of the most abundant sulfur-containing organic compounds in nature (Benning, 1998), along with glutathione and the amino acids cysteine and methionine, and therefore its complete biodegradation (mineralization) plays an important role in the biogeochemical sulfur and carbon cycles (Harwood and Nicholls, 1979). SQ is part of the green vegetable diet of herbivores and omnivores, and it can be a select nutrient of prominent bacteria and a source of hydrogen sulfide (H_2_S) in the human gastrointestinal tract through strictly anaerobic bacterial metabolism (Hanson et al., 2021; Burrichter et al., 2018).

To date, five different principal pathways and biochemical strategies, as well as several pathway variants (as detailed below), have been uncovered for the utilization of SQ as carbon and energy source in heterotrophic, aerobic or anaerobic bacteria; four of these strategies can be referred to as sulfoglycolytic pathways and one as sulfolytic pathway (see below) (Burrichter et al., 2018; Liu et al., 2020, 2021; Roy et al., 2003; Snow et al., 2021; Ye et al., 2023; Wei et al., 2022; Felux et al., 2015; Li et al., 2020; Chu et al., 2023; Sharma et al., 2022; Denger et al., 2014; Frommeyer et al., 2020). The first sulfoglycolytic pathway was described for *Escherichia coli* and is found in a wide range of other Enterobacteria. This pathway is analogous to the Embden-Meyerhof-Parnas (EMP) pathway for glucose-6-phosphate and can therefore be referred to as the sulfo-EMP pathway (Liu et al., 2021; Wei et al., 2022; Denger et al., 2014; Sharma et al., 2021). Another pathway was initially described for a *Pseudomonas* strain and is analogous to the Entner-Doudoroff (ED) pathway, namely the sulfo-ED pathway (Felux et al., 2015; Li et al., 2020). Two more types of sulfoglycolytic pathways in aerobic and anaerobic bacteria—for which a new, bifurcated pathway variant is the subject of the present study—employ enzyme reactions analogous to the pentose phosphate pathway (PPP), the 6-deoxy-6-sulfofructose transaldolase (sulfo-TAL) (Liu et al., 2020; Frommeyer et al., 2020; Snow et al., 2023) and the 6-deoxy-6-sulfofructose transketolase (sulfo-TK) (Liu et al., 2021; Chu et al., 2023) pathways. Moreover, sulfolytic mono- and dioxygenase pathways have been demonstrated for aerobic bacteria (Liu et al., 2021, 2023; Ye et al., 2023; Sharma et al., 2022), in which SQ is desulfonated in the first reaction step by alkanesulfonate-type SQ mono- or dioxygenases; the resulting 6-dehydroglucose is oxidized to glucose and funneled into ‘normal’ glycolysis. The latter are the only known pathways through which SQ is completely degraded in a single bacterial strain, whereas for all other known pathways, only primary degradation of SQ is achieved: no desulfonation reactions take place, but C_3_-organosulfonates (2,3-dihydroxypropanesulfonate (DHPS) or 3-sulfolactate (SL)) (Liu et al., 2020, 2021; Wei et al., 2022; Felux et al., 2015; Li et al., 2020; Denger et al., 2014; Frommeyer et al., 2020; Sharma et al., 2021) or C_2_-organosulfonates (2-hydroxyethanesulfonate (isethionate, ISE) (Liu et al., 2021) or sulfoacetate (SAc) (Chu et al., 2023)) are excreted as degradation products by these organisms. These products can be degraded by other bacteria. Therefore, in these instances, SQ is mineralized by two-member aerobic or anaerobic consortia, through which the sulfonate moiety is ultimately released as either sulfate (SO_4_^2−^), sulfite (SO_3_^2−^), or hydrogen sulfide (H_2_S) (Burrichter et al., 2018; Wei et al., 2022; Denger et al., 2012, 2014; Peck et al., 2019). Of relevance to the present study is a pathway for the utilization of ISE by the strictly anaerobic bacterium *Bilophila wadsworthia* as a sulfite donor for anaerobic respiration, producing sulfide and acetate (Peck et al., 2019).

**Figure 1:**
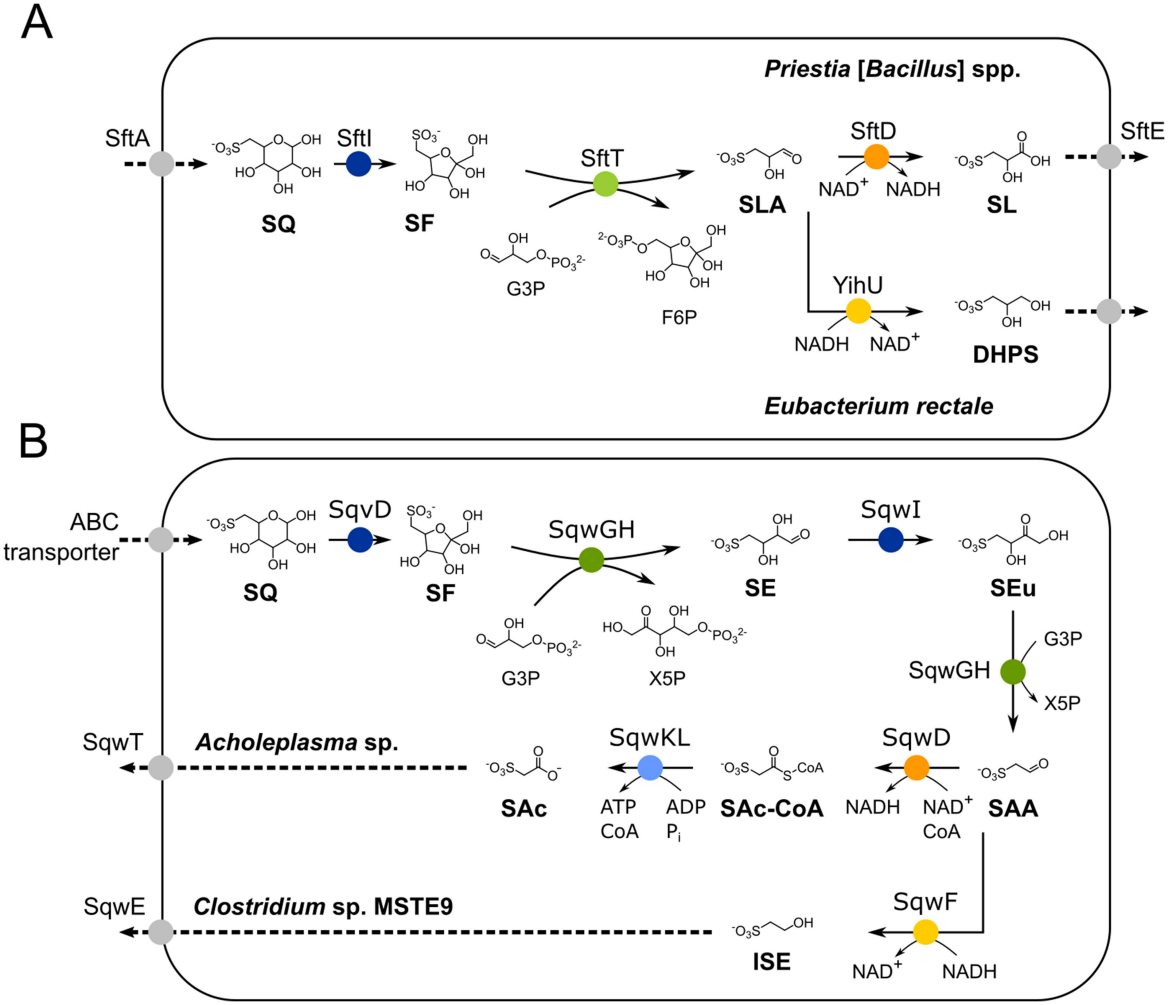
**(A)** Illustration of the known 6-deoxy-6-sulfofructose transaldolase (sulfo-TAL) pathway. SQ is imported, most likely by transporter SftA, and isomerized to 6-deoxy-6-sulfofructose (SF) by the SQ isomerase SftI. The non-sulfonated C_3_-(glycerone) moiety of SF is transferred to glyceraldehyde 3-phosphate (G3P) by the SF transaldolase SftT, resulting in fructose-6-phosphate (F6P) and 3-sulfolactaldehyde (SLA); the former is used for energy generation and regeneration of the G3P acceptor molecule (not shown, see Frommeyer et al., 2020 for details). The SLA is oxidized to 3-sulfolactate to generate additional NADH for aerobic respiration in aerobic *Priestia* [formerly *Bacillus*] *aryabhattai* SOS1, whereas in strictly anaerobic *Clostridium* spp. and *Eubacterium rectale* it is employed as internal electron acceptor for NAD^+^ regeneration (fermentation) and reduced to 2,3-dihydroxypropane-1-sulfonate (DHPS); both degradation products are not further converted but excreted (Frommeyer et al., 2020). **(B)** Illustration of the two major 6-deoxy-6-sulfofructose transketolase (sulfo-TK) pathway variants. SQ is imported, most likely by an ABC transporter (Arumapperuma et al., 2024), and isomerized to SF by isomerase SqvD (Liu et al., 2021; Chu et al., 2023). A non-sulfonated C_2_-(ketol) moiety of SF is transferred to G3P by SF transketolase (SqwGH), yielding xylulose-5-phosphate (X5P) and the sulfo-sugar 4-deoxy-4-sulfoerythrose (SE). The SE is then isomerized to 4-deoxy-4-sulfoerythrulose (SEu) by the SEu isomerase (SqwI), and the SEu is converted by SqwGH to yield another molecule of X5P and 2-sulfoacetaldehyde (SAA). In *Clostridium* sp. MSTE9 (Liu et al., 2021), the SAA is reduced to isethionate (ISE; 2-hydroxyethanesulfonate) by the NADH-dependent SAA reductase SqwF, which is then excreted (SqwE). Another variant of this pathway has been described for *Acholeplasma* sp. using recombinant enzymes (Chu et al., 2023), in which SAA is oxidized further to 3-sulfoacetate (SAc) via a NAD^+^-dependent, CoA-acylating sulfoacetaldehyde dehydrogenase (SqwD), producing sulfoacetyl-CoA (SAc-CoA), and a CoA ligase (SqwKL); the SAc is exported by the exporter SqwE. Isomerases are indicated in blue, reductases in yellow, dehydrogenases in orange, ligase in light blue, transaldolase in light green, and transketolase in dark green. Importers and exporters are indicated in grayscale.

For the primary degradation of SQ via the sulfo-TAL (transaldolase) pathway, two pathway variants have been demonstrated in aerobic *Priestia* [formerly *Bacillus*] spp. and anaerobic *Clostridium* spp. and *Eubacterium rectale* strains (Liu et al., 2020; Frommeyer et al., 2020), respectively. They initially follow the same reaction steps (Figure 1A), in which SQ is isomerized to 6-deoxy-6-sulfofructose (SF) by SQ isomerase (SftI), before a non-sulfonated C_3_-unit is transferred by SF transaldolase SftT from SF to an acceptor molecule, for example to glyceraldehyde-3-phosphate (G3P), yielding fructose-6-phosphate (F6P) to drive energy generation and growth. The organosulfonate remaining is 3-sulfolactaldehyde (SLA), and the sulfo-TAL pathway variants determine how the SLA is further utilized (Figure 1A): In the aerobic (oxidative) variant, SLA is further oxidized to SL by a NAD^+^-dependent SLA dehydrogenase (SftD), thus yielding additional electrons for aerobic respiration and ATP synthesis, while in the anaerobic (reductive) variant, the SLA is reduced to DHPS by NADH-dependent reductases (e.g., YihU), thus recovering NAD^+^ as electron acceptor in a fermentation step. Notably, this fermentation step is also a component of the sulfo-EMP pathway of facultative *E. coli* (Denger et al., 2014), while an oxidative variant of the sulfo-EMP pathway exists that produces SL (Burrichter et al., 2018; Liu et al., 2021). In addition, a third variant of the sulfo-TAL (transaldolase) pathway exists in *Enterococcus gilvus*, in which the intermediate DHPS is dehydrated and oxidized to 3-sulfopropionaldehyde. The 3-sulfopropionaldehyde can then either be reduced to 3-hydroxysulfopropionate, or oxidized to 3-sulfopropanoate by a NAD^+^-dependent, CoA-acylating dehydrogenase and an ATP-dependent sulfopropanoate-CoA ligase, hence yielding additional ATP (Chen et al., 2024) (not shown in Figure 1).

There are also two known variants of the sulfo-TK (transketolase) pathway which were uncovered by genome mining and demonstrated through recombinant enzymes. These variants also follow the same reaction steps initially (Figure 1B). First, SQ isomerase (SqvD) produces SF, from which a non-sulfonated C_2_-unit (ketol moiety) is transferred to G3P by transketolases (SqwGH), yielding xylulose-5-phosphate (X5P) to drive energy generation and growth. The remaining sulfonated product is 4-deoxy-4-sulfoerythrose (SE). It is converted by another isomerase (SqwI) to 4-deoxy-4-sulfoerythrulose (SEu), from which again the C_2_-(ketol) moiety is transferred to G3P by the transketolase enzyme (SqwGH), yielding another X5P. The organosulfonate remaining from this second transketolase reaction is 2-sulfoacetaldehyde (SAA), and the sulfo-TK pathway variants determine how the SAA is further utilized (Figure 1B). In the oxidative variant described for aerobic *Acholeplasma* sp. (Chu et al., 2023), the SAA is converted to SAc by a NAD^+^-dependent, CoA-acylating SAA dehydrogenase (SqwD) and an ATP-dependent sulfoacetate-CoA ligase (SqwKL), hence, yielding additional ATP. In the reductive variant described for the anaerobic *Clostridium* sp. MSTE9 (Liu et al., 2021), SAA is converted to ISE by a NADH-dependent reductase (SqwF) by which NAD^+^ is recovered.

Here, we report on the enrichment and characterization of a strictly anaerobic SQ-degrading consortium that produces ISE as the main product, but also DHPS with concomitant production of acetate and molecular hydrogen (H_2_), and that can degrade ISE to hydrogen sulfide in a second step. Through growth experiments, analytical chemistry, genomics, proteomics, and transcriptomics, we found evidence for a combination of the sulfo-TK and sulfo-TAL pathways in the primary SQ-degrading bacterial member of the consortium, while the glycyl-radical enzyme pathway as described for *Bilophila wadsworthia* (Peck et al., 2019; Xing et al., 2019) was identified for the ISE-degrading member of the consortium. Furthermore, a newly identified gene cluster for SQ utilization was also found in the isolate *Faecalicatena* sp. DSM22707, which grew by SQ fermentation in pure culture and produced mainly ISE but also SL and SLA, in addition to acetate, butyrate, succinate, and formate, but not H_2_. We then reproduced the degradation of SQ by the consortium using a defined co-culture model, consisting of *Faecalicatena* sp. DSM22707 and *Bilophila wadsworthia* 3.1.6. Our findings provide the first description of an additional, bifurcated SQ pathway in anaerobic fermenting bacteria of the family Lachnospiraceae, one of the most abundant taxa in the human gut and rumen microbiota. Furthermore, we expand on the knowledge of the diversity of pathways of sulfidogenic SQ degradation by strictly anaerobic co-cultures, comprising SQ-fermenting bacteria and cross-feeding of the SQ-degradation intermediate to organosulfonate-respiring and H_2_S-producing organisms, a process which is also relevant to gut microbiomes, since H_2_S is a metabolite that can exert manifold beneficial and harmful impacts on the host (Banerjee, 2011).

## 2 Materials and methods

### 2.1 Chemicals

All routine chemicals were purchased from Sigma Aldrich (Darmstadt, Germany), Carl Roth (Karlsruhe, Germany), or Merck (Darmstadt, Germany). Sulfoquinovose (SQ) and racemic 3-sulfolactate (SL) were purchased from MCAT GmbH (Donaueschingen, Germany). Racemic 2,3-dihydroxypropanesulfonate (DHPS) was synthesized as described previously (Mayer et al., 2010). Isethionate (2-hydroxyethanesulfonic acid sodium salt) was purchased from Merck Schuchardt (Hohenbrunn, Germany).

### 2.2 Software

Plots to illustrate data such as growth curves were generated with Origin, Version 2021b (OriginLab Corporation, Northampton, MA, United States). Plots were aligned and figures were arranged using Inkscape 1.3.^1^ Homology predictions between protein sequences or nucleotide sequences were performed using BLAST (basic local alignment search tool) (Altschul et al., 1990) with default options. For the nucleotide BLAST, uncultured bacteria and models were excluded unless otherwise noted.

### 2.3 Cultivation of enrichment cultures and bacterial isolates

The mixed culture described here was enriched with 10 mM SQ as the sole source of carbon and energy under strictly anoxic conditions, using anoxic, iron(II)-sulfide-rich (black) sediment from Lake Constance as inoculum, as sampled with a sediment gravity corer in October 2014 from Mainau Bay (*Obere Güll*; 47°42′00.0"N, 9°11′31.6"E); the sediment surface at approximately 22 m water depth was oxic and the sample was taken from approximately 15 cm deep in the sediment. After four transfers, SQ was confirmed to be degraded and isethionate was detected as intermediate, accompanied by the growth of bacteria visible as turbidity and microscopically. A stable, highly enriched mixed culture was achieved by repeated sub-cultivation from liquid to liquid cultures, as well as by several rounds of anoxic agar dilution series and picking of colonies, each with SQ as the sole source of carbon and energy (Pfennig, 1978); the consortium has since been routinely maintained by transfers at weekly or monthly intervals. The mineral salt medium used was the carbonate-buffered freshwater medium described by Widdel and Pfennig (1981) containing 1.0 g NaCl, 0.4 g MgCl_2_ × 6 H_2_O, 0.2 g KH_2_PO_4_, 0.25 g NH_4_Cl, 0.5 g KCl, 0.15 g CaCl_2_ × 2 H_2_O per 1 L double distilled water (ddH_2_O). As a redox indicator, 1 mL of a 0.4 mg/mL Na-resazurin solution was added. The medium was autoclaved for 30 min at 121°C. Then, 50 mM bicarbonate (final concentration) buffer was added and the medium was reduced with 1 mM titan-(III)-nitrilotriacetate [final concentration (Moench and Zeikus, 1983)]. As an additional sulfur source, 200 μM Na_2_S was added (final concentration). 1 mL trace element solution SL10 (DSMZ medium 141), 1 mL selenite-tungstate solution (DSMZ medium 385), and 0.5 mL 7-vitamin solution (Widdel and Pfennig, 1981) were also added. Finally, the pH was adjusted to 7 with 1 M HCl and the medium was distributed into sterile serum flasks or culture tubes, as appropriate.

The initial enrichment culture, its sub-cultivations, and the ultimately obtained highly enriched mixed culture (consortium) were grown with SQ in anoxic Hungate-type tubes or serum bottles (up to 50 mL) sealed with butyl rubber stoppers. The consortium was later also grown in triplicate in 125 mL flasks for growth experiments and DNA extraction for metagenomics and Illumina amplicon sequencing. For total-protein and total-RNA preparations for metaproteomics and metatranscriptomics, respectively, the consortium was grown in triplicate in 500 mL flasks. All cultures were incubated at 28°C in the dark without shaking. Cultures in which the headspace (N_2_/CO_2_, 80%/20%) was exchanged for H_2_/CO_2_ (79%/21%) were incubated horizontally, in order to increase the surface area available for gas exchange.

*Faecalicatena* sp. DSM22707 was purchased from the Leibniz-Institute DSMZ – German Collection of Microorganisms and Cell Cultures GmbH (Braunschweig, Germany) and maintained in a complex medium based on DSMZ medium 339a, containing 5 g tryptone/peptone, 5 g yeast extract, 0.5 g NaHCO_3_, 0.5 mL Na-resazurin (stock, 0.4% w/v), 10 mL salt solution (0.25 g CaCl_2_ × 2 H_2_O, 0.50 g MgSO_4_ × 7 H_2_O, 2.00 g NaCl dissolved in 500 mL ddH_2_O), 5 mL phosphate buffer (1.00 g K_2_HPO_4_, and 1.00 g KH_2_PO_4_ in 500 mL ddH_2_O), 10 mL L-cysteine solution (0.25 g L-cysteine-HCl x H_2_O/10 mL ddH_2_O), 0.5 mL vitamin K_1_ solution (100 mg/L, DSMZ Medium 78) and 5 mL hemin solution (0.5 mg/mL, DSMZ Medium 78) per 1 L ddH_2_O. Phosphate buffer, L-cysteine, vitamin K_1,_ and hemin solutions were added after autoclaving. The pH was adjusted to 7.0 by the addition of 0.5 M Na_2_CO_3_ or 1 M HCl. For growth experiments with 10 mM SQ as substrate, strain DSM22707 was transferred to Hungate-type tubes containing the freshwater medium as described above, supplemented by 0.05% YE, and by hemin and vitamin K_1_ at concentrations as in DSMZ medium 339a. For differential proteomics experiments, 10 mM glucose served as an alternative carbon and energy source for strain DSM22707.

Growth was monitored by measuring optical density (OD) at 600 nm in samples taken from the cultures in plastic cuvettes using a Pharmacia Novaspec II spectrophotometer (LKB Biochrom, United Kingdom) or directly in Hungate tubes using a Camspec M107 spectrophotometer (Leeds, UK). Substrate turnover and product formation were monitored by HPLC, GC, or LC–MS (see below).

### 2.4 Microscopy

Cultures were examined by phase-contrast microscopy using a ZEISS Axiophot (Jena, Germany) microscope and a 1.3 EC Plan-NEOFLUAR objective at either 40× or 100× magnification; the microscope was equipped with an Axiocam 305 color camera. Images were acquired using AxioVision software release 4.7.2. Images were processed using Fiji Image J 1.52p (Schneider et al., 2012). Cells were embedded on agarose-coated slides, prepared according to the method described by Pfennig and Wagener (1986), but instead of agar, a 2% agarose solution was dispersed on clean microscope slides; the agarose slides were dried overnight in a dust-free box prior to use.

### 2.5 Analytical methods

#### 2.5.1 High-pressure liquid chromatography (HPLC)

Acetate, other short-chain fatty acids, or other potential fermentation products were separated, screened and, if appropriate, identified and quantified against authentic standards, by ion exchange chromatography using a Shimadzu HPLC LC-20 system coupled to a refractive index detector (RID), as described by Keller et al. (2019), except for the eluent concentration, which was 30 mM H_2_SO_4_. Chromatograms were analyzed using LabSolutions version 5.97 SP1 (Shimadzu Corporation, Duisburg, Germany).

#### 2.5.2 Liquid chromatography–mass spectrometry (LC–MS) and high resolution—heated electrospray ionization—tandem mass spectrometry (HR-HESI-MS/MS)

For routine LC–MS analysis, a Shimadzu LCMS-2020 single quadrupole mass spectrometer system with an electrospray ionization (ESI) ion source coupled to a tandem LC-40B XR pump, a SIL-40C XR autosampler and a CTO-40S column oven (30°C) was used. A HILICON iHILIC-Fusion(+) column (100 × 2.1 mm, 3.5 μm, 100 Å) was used for the separation of the organosulfonates. Mobile phase A consisted of 10 mM ammonium formate plus 10% (v/v) acetonitrile (HPLC grade, Sigma Aldrich) and 90% (v/v) MilliQ water, and mobile phase B consisted of 10 mM ammonium formate and 90% (v/v) acetonitrile plus 10% (v/v) MilliQ water. The flow rate was 0.3 mL min^−1^. The gradient started at 100% B for 2 min, and then B was decreased to 0% within 10 min and held at 0% B for 1 min; then, it was increased back to 100% B within 1 min, and the column was re-equilibrated for 6 min at 100% B. The MS was operated in the negative ion mode with the following settings: ionization, −3.5 kV; interface current, 0.2 μA; temperature, 200°C; entrance lens voltage, 20.0 V; conversion dynode voltage, 10.0 kV; detector voltage, 1.00 kV; nebulizing gas flow, 1.50 L min^−1^; drying gas flow, 15.00 L min^−1^. The analytes were detected by their mass-to-charge ratio (*m/z*) of the quasimolecular ion [M-H]^−^, which were 125 for ISE, 155 for DHPS, 169 for SL, and 243 for SQ. Moreover, the retention times of the compounds were compared to authentic standards for identification. ISE and SQ were quantified by LC–MS using calibration curves obtained by measuring known concentrations between 0.1 and 1 mM of the respective standards. Peak areas were normalized to the area of an internal organosulfonate standard that was contained in all samples, *p*-toluenesulfonate sodium salt (TS; 0.2 mM final concentration) as detected at *m*/*z* 177. All data were recorded and analyzed using the Shimadzu LabSolutions software (version 5.97). Samples of culture fluid for LC–MS analysis were prepared as follows: the culture fluid was centrifuged at 10.000×*g* for 5 min at 4°C; the supernatant was diluted 1:10 with MilliQ water, except for samples for which DHPS and SL were to be detected, and then mixed at a ratio of 7:3 (v/v) with ≥99.9% acetonitrile containing 6.7 mM TS as internal standard; these TS-spiked samples were centrifuged again and the supernatant was analyzed.

HR-HESI-MS/MS measurements of spent medium samples of *Faecalicatena* sp. DSM22707 were performed using a Thermo Fisher Scientific LTQ Orbitrap XL mass spectrometer with a HESI II ion source coupled to a Dionex Ultimate 3,000 UHPLC system equipped with a Phenomenex Kinetex HILIC column (150 × 2.1 mm, 2.6 μm), operated at a flow rate of 0.25 mL min^−1^. Ammonium acetate buffer (50 mM, pH 5) containing 10% acetonitrile served as solvent A. Solvent B was acetonitrile containing 0.1% (v/v) acetic acid. HPLC separation conditions were 100% B for 2 min, within 18 min to 100% A, 100% A for 5 min, back to 100% B in 0.5 min, and 4.5 min re-equilibration at 100% B. The LTQ Orbitrap XL MS was operated in negative ion mode with a resolution setting of 100,000. The instrument was freshly calibrated prior to the measurements. Tandem mass spectra (MS/MS) of the [M-H]^−^ quasimolecular ions were recorded using the LTQ ion trap detector.

For derivatization of oxo-group-containing SQ degradation intermediates the acidified supernatant (pH 3) of SQ-grown *Faecalicatena* sp. DSM22707 was incubated with 2,3,4,5,6-pentafluorobenzylhydroxylamine (PFBHA) at a final concentration of 2 mM at room temperature overnight. Acetonitrile was added to the samples at 50% (v/v) to precipitate the proteins. After an incubation of 1 h, the samples were centrifuged at 21,000×*g* and the supernatant was subjected to HR-HESI-MS/MS. Some samples were extracted after acidification to pH 1 with 2 N HCl with ethyl acetate. The ethyl acetate extracts were concentrated in a gentle nitrogen stream and redissolved in 20 μL methanol for analysis.

#### 2.5.3 Gas chromatography (GC) for detection of H_2_ and CH_4_ in culture headspace

H_2_ was quantified, and CH_4_ was measured to confirm its absence in the gas phase of the cultures after growth, using an SGI 8610C GC (SRI Instruments, Los Angeles, CA, United States) according to the method described by Keller et al. (2019). Briefly, the GC was equipped with a thermal conductivity detector (150°C) for the detection of H_2_ and a flame ionization detector (135°C) for the detection of CH_4_. A Hayesep-D column (3 m, maintained at 60°C) was used for the separation of gases, with N_2_ as the carrier gas (25 psi). Chromatograms were recorded and analyzed using the PeakSimple v4.44 software.

#### 2.5.4 Sulfide assay

Sulfide was quantified as described by Cline (1969), with volumes adjusted for small sample sizes (Thiel et al., 2019). Absorbance at 670 nm was measured using a HITACHI U-1100 spectrophotometer. Samples for sulfide quantification were taken in biological quadruplicates (*n* = 4) from cultures grown in Hungate-type tubes and fixed by the addition of zinc chloride (Cline, 1969) prior to storage until analysis.

### 2.6 Molecular methods and bioinformatics

#### 2.6.1 DNA extraction, (meta)genomic, and Illumina amplicon sequencing

The total DNA of the consortium was extracted from samples of cells harvested in the exponential growth phase according to the ‘Bacterial DNA isolation CTAB’ (cetrimonium bromide) protocol (version: 11/12/2012) of the JGI (DOE Joint Genome Institute) (William and Helene Feil, 2012) with the following exception: the cell suspensions were incubated with 0.1 mg mL^−1^ of proteinase K for 3 h prior to the addition of lysozyme, which resulted in a more efficient cell lysis, as verified by microscopy. After the addition of lysozyme, the protocol was followed without additional changes.

Total DNA from *Faecalicatena* sp. DSM22707 was extracted using the Monarch Genomic DNA Purification Kit (New England Bio Labs Inc., Germany), following the protocol for Gram-positive bacteria and archaea, with the following exception: the lysis step was increased to 30 min, the proteinase K step was extended to 60 min, and the RNase step was performed for 30 min; the purification column was incubated with elution buffer for 10 min prior to centrifugation for DNA elution.

For the metagenomic sequencing of the consortium, total DNA was submitted to Eurofins Genomics (Konstanz, Germany) using the Illumina platform with 2× 150 bp paired-end reads. The quality of the paired-end sequencing reads was assessed using FastQC v0.11.9 (Andrew, 2015), followed by quality score-based filtering of low-quality reads based on Minoche et al. (2011) using the lllumina-utils v2.11 codebase (Eren et al., 2013, 2021). After quality filtering, >80% of the initial 47 million read pairs were retained. The quality-filtered reads were assembled using MetaSPAdes v3.15.3 (Nurk et al., 2017). Contigs with a minimum size of 1,000 bp were submitted to JGI’s Integrated Microbial Genomes (IMG) GOLD pipeline for functional annotation (Mukherjee et al., 2020). Quality-filtered reads were mapped to the assembled contigs using Bowtie2 v2.3.5.1 (Langmead and Salzberg, 2012) and Samtools v1.7 (Danecek et al., 2021). Open reading frames were identified using Prodigal v2.6.3 (Hyatt et al., 2010). Anvi’o v7 (Eren et al., 2021) was used to identify single-copy core genes, and to determine taxonomy based on The Genome Taxonomy Database [GTDB v95 (Parks et al., 2018, 2022)], using the DIAMOND sequence aligner v0.9.14 (Buchfink et al., 2015). Initial binning of contigs was performed using MetaBAT2 v2:2.15 (Kang et al., 2015) for contigs with a minimum length of 1,500 bp. To reconstruct metagenome-assembled genomes (MAGs), the resulting bins were manually refined using Anvi’o, based on contig features such as coverage, GC, and tetranucleotide content. Identified ribosomal genes did not follow the coverage pattern of the bins and were assigned based on taxonomic classification using the rdp classifier (16S rRNA training set 18) (Wang et al., 2007) and/or NCBI BLAST (refeq_rna database) (O'Leary et al., 2016), which allowed for assignment to the bins based on results matching the GTDB classification. The quality of the recovered MAGs was assessed using Anvi’o v7 (Eren et al., 2021) based on the completeness and redundancy of conserved single-copy core genes from closely related taxa.

To determine the genome sequence of *Faecalicatena* sp. DSM22707, total DNA was submitted to Eurofins Genomics (Konstanz, Germany) for sequencing using the Illumina platform with 2× 150 bp paired-end reads. The quality of the obtained reads was assessed using FastQC, followed by quality filtering using lllumina-utils, as described above; 92.7% of reads were retained from >5.4 million reads. Reads were assembled into contigs using SPAdes v3.15.4 (Prjibelski et al., 2020) and the quality was assessed with checkM v1.0.8 (Parks et al., 2015) and QUAST (Quality Assessment Tool) v4.4 (Gurevich et al., 2013) on the KBase web server (Arkin et al., 2018). All contigs >500 bp were submitted to JGI’s Integrated Microbial Genomes (IMG) GOLD pipeline for functional annotation. The draft genome (IMG Taxon ID 2956855026), with a size of 4.8 Mb and a GC content of 42.03%, has an estimated completeness of 98.83% with an estimated contamination of 0.68%. It was annotated to encode 4,574 protein-coding genes and three 16S rRNA gene copies of 343, 735, and 522 bp, and three 23*S* rRNA gene copies of 1,353, 897, and 575 bp.

For Illumina amplicon sequencing, the INVIEW Microbiome Profiling from Eurofins Genomics (Konstanz, Germany) was used, for which the total extracted DNA of the consortium was submitted (see above); primers used for amplification of the 16S V3 – V5 region were (forward) 5’-CCTACGGGNGGCWGCAG-3′ (Herlemann et al., 2011) and (reverse) 5’-CCGYCAATTYMTTTRAGTTT-3′ (Quince et al., 2011). The amplicons were sequenced using Illumina MiSeq, with 2 × 300 bp paired-end reads, resulting in 60,000 read pairs. The downloaded reverse and forward reads were merged with NGmerge v0.3 (Gaspar, 2018) (minimum overlap, 5; allowed mismatches 0.05), and trimmed with Trimmomatic v0.40 (Bolger et al., 2014) (sliding window, 4; average phred quality, 15; minimum length, 500). Quality was checked with FastQC v0.11.9 (Andrew, 2015). All further steps were performed as described by Fournier et al. (2021). Briefly, for denoising and taxonomic affiliation, DADA 2 (Callahan et al., 2016) and the q2-vsearch tool of QIIME 22019.10 (Bolyen et al., 2019) were used, respectively. The SILVA database Release 138 (Glöckner et al., 2017; Quast et al., 2013; Yilmaz et al., 2014) was chosen as the reference database.

#### 2.6.2 mRNA sequencing and total proteomics

The consortium was grown in triplicate and harvested in the exponential phase. Each triplicate was subjected to mRNA sequencing and total proteomics. For the differential proteomics study of *Faecalicatena* sp. DSM22707, cultures were grown each in the presence of 0.05% YE and either 10 mM D-(+)-glucose (*n* = 3) or 10 mM SQ (*n* = 3) and harvested at the end of exponential phase by centrifugation (15,557×*g*, 4°C, 15 min). For samples subjected to RNA extraction, RNA*late*r Tissue Collection: RNA Stabilization Solution (Ambion Life Technologies, Carlsbad, United States) was added according to the manufacturer’s instructions. All harvested cell pellets were stored at −80°C until further processing.

For total proteomics, cells were lysed by sonication of the resuspended cells in 1 mL TE buffer (10 mM Tris, 1 mM EDTA, pH 8.0) with a UP50H—Compact Lab Homogenizer, Hielscher Ultrasound Technology (Teltow, Germany), operated at cycle 0.5 and amplitude 100%. The sonotrode was lowered into the cooled tube. Activation and pause alternated for 30 s each for a total of 10 min. Cell disruption was confirmed by microscopy. Cell debris was removed for samples of *Faecalicatena* sp. DSM22707 by centrifugation (10,000×*g*, 4°C, 5 min). After the determination of protein concentration by a Bradford assay (Bradford, 1976), samples were adjusted with TE buffer to equal protein concentrations and then submitted to the Proteomics Centre of the University of Konstanz,^2^ where total proteomic analysis (long LC gradients) was performed as previously described (Burrichter et al., 2018). The Proteome Discover software (Thermo Fisher Scientific, Waltham, MA, United States) or the MASCOT software (Matrix Science, Boston, MA, United States) was used to match peptide fingerprints against a local database containing all metagenome- or genome-encoded candidate enzymes (cutoff ≥1,000 bp), respectively, as derived from the IMG annotations (see above). For each identified protein, the peak area values of all identified peptides were used to calculate mean areas with standard deviation for all the proteins identified for the consortium. For *Faecalicatena* sp. DSM22707, a label-free quantification (LFQ) approach was used by implementing the DEP package (Zhang et al., 2018). To identify significantly upregulated proteins, we chose a log_2_ fold change cutoff of 3 and a significance value (*p*-value) cutoff of 5×10^−5^, as the fraction of differentially expressed proteins should be around 10–15% (Zhang et al., 2018). Volcano plots were generated using the EnhancedVolcano package (Blighe et al., 2022), both packages were run in R v4.2.0 (R Core Team, 2022) using the graphical interface RStudio v2022.2.3.492 (RStudio Team, 2022). Gene clusters were generated and aligned using cblaster v1.3.18 (Gilchrist et al., 2021) or clinker v0.0.28 (Gilchrist and Chooi, 2021).

For total RNA extraction, the cell suspensions were thawed at room temperature. The RNAlater solution was removed by adding 1.5 mL of extraction buffer (50 mM Na-acetate, 10 mM EDTA, pH 4.2) for dilution, and centrifugation (15,557×*g*, 30 min, 4°C) and discarding of the supernatant; the samples were kept on ice. The RNA preparation was done using a protocol based on phenol/chloroform extraction, as described by Schneider et al. (2017) including modifications from Klotz et al. (2022). These RNA samples were submitted to Eurofins Genomics for rRNA depletion, cDNA synthesis, and 150 bp paired-end sequencing, when following their in-house routine protocols. The resulting sequences were quality trimmed and adapters were removed using Trimmomatic (v0.40) (Bolger et al., 2014) (seed mismatches: 4, palindrome clip threshold: 30, simple clip threshold: 10, SLIDINGWINDOW:4:20 and MINLEN:40). Quality was checked using FastQC v0.11.9 (Andrew, 2015). Transcripts were pseudo-aligned and quantified against the coding genes of the metagenome from the consortium (see above) using kallisto v0.46.0 (Bray et al., 2016).

#### 2.6.3 Enzyme structure prediction

3D models of *Faecalicatena* sp. DSM22707 transketolase (domains: F1, IMG Gene ID: 2956859247; F2, IMG Gene ID: 2956859248) and *Clostridium* sp. MSTE9 (Cl1, IMG Gene ID: 2524472430; Cl2, IMG Gene ID: 2524472431) were predicted using Alphafold (v. 2.3.2) (Jumper et al., 2021) in multimer mode to obtain a ⍺2β2 heterotetramer resembling *Scheffersomyces stipitis* (PDB code: 5XU2). Models for *Faecalicatena* sp. DSM22707 transaldolases (F1, IMG Gene ID: 2956859250, F2, IMG Gene ID 2956859251) were done in monomer mode resembling *Priestia* [*Bacillus*] *megaterium* 6-deoxy-6-sulfofructose transaldolase (BmSF-TAL, PDB code: 8 BC4, UniProtKB D5E1T2) (Snow et al., 2023). All models were calculated locally on a customized computer server at the University of Konstanz. The template database included Protein Data Bank entries up to August 11, 2023, and structural analyses and figures were generated in Chimera.^3^ Root mean square deviation (RMSD) values for C⍺ atoms between the predicted models and the corresponding crystal structures were calculated to indicate the structural agreement of the predictions. Aligned to 5XU2, the model of transketolase F1 had an RMSD_C⍺_ = 0.853 Å for 225 aligned residues (out of a total of 319 amino acids) and F2 had an RMSD_C⍺_ = 1.176 Å for 196 aligned residues (out of a total of 274 amino acids). The RMSD_C⍺_ values of F1 and F2 transketolase to 6YAK were 0.672 Å for 262 aligned amino acids and 0.888 Å for 281 amino acids, respectively. The RMSD_C⍺_ of transaldolase F1 to 8 BC4 was 0.759 Å for 209 atom pairs and 0.925 Å for 140 atom pairs for F2.

## 3 Results

### 3.1 Enrichment of a strictly anaerobic, stable mixed culture (consortium) that degrades SQ to isethionate and analysis of its phylogenetic composition

**Figure 2:**
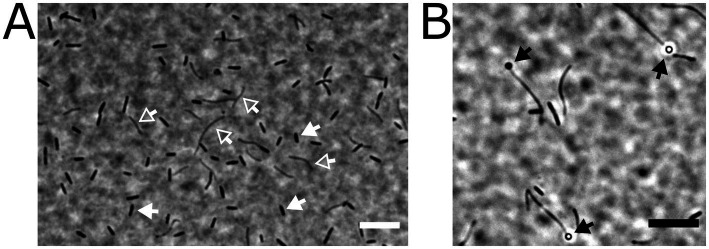
Microscopic appearance of two different cell types present in the highly enriched, stable anaerobic consortium that degraded SQ via ISE to H_2_S. The cells were embedded on agarose-coated slides and imaged by phase-contrast microscopy. The two different cell types are indicated by arrows, rod-shaped cells (indicated by white-filled arrows) and filamentous cells (white open arrows) (**A**). In some instances, the filamentous cells carried terminal spores (black arrows in panel **B**). Scale bars, 10 μm.

We established several bacterial enrichment cultures with chemically synthesized SQ as the sole carbon and energy source for both aerobic (e.g., Felux et al., 2015; Frommeyer et al., 2020; Denger et al., 2012) and strictly anaerobic heterotrophic growth. For one of the latter, we provided 10 mM SQ in a titanium(III)-nitrilotriacetate-reduced freshwater mineral salt medium without any additional electron acceptor (except CO_2_), and inoculated it with an iron(II)-sulfide-rich (black) sediment sample from Lake Constance. The culture remained active after several transfers and, interestingly, did not convert SQ to the C_3_-intermediates DHPS or SL, but to the C_2_-intermediate ISE, which was new at that time. Furthermore, it produced H_2_S (and no methane), suggesting that ISE was further utilized for organosulfonate respiration in the enrichment culture (see below). During subcultivation, the enrichment culture was subjected to agar-shake dilution in several stages with SQ as substrate, in an attempt to isolate the SQ-degrading community member; however, when individual colonies were picked back into SQ-liquid medium, mixed cultures were always obtained, as visible by microscopy, consisting of rod-shaped and filamentous cells (Figure 2). The rod-shaped organisms appeared to be the most abundant, and the filamentous bacterium appeared to produce terminal spores (Figure 2). Since our many attempts to isolate the primary SQ-degrading, ISE-producing strain were not successful, we proceeded to characterize the consortium by molecular methods.

**Figure 3:**
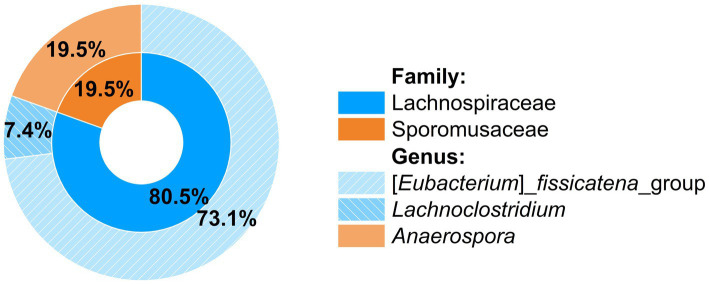
Phylogenetic composition of the SQ-degrading consortium as determined by Illumina amplicon sequencing of a 570 bp fragment spanning the V3 to V5 region of the 16S rRNA gene. Relative abundances were calculated from the sequence reads obtained after quality assessment. Only three genera were detected within two families.

The phylogenetic composition of the consortium was first examined by Illumina amplicon sequencing of the V3 to V5 region of the 16S rRNA gene, which allowed for the assignment of amplicon sequence variants (ASVs) up to the genus level and for calculation of relative abundances (Figure 3). Contributing 73.1% of the total reads was an ASV affiliated to the [*Eubacterium*] *fissicatena_*group (genus *Faecalicatena*) within the family Lachnospiraceae of the phylum Bacillota [Firmicutes], a SILVA 138 database (Gaspar, 2018) group with 371 sequence entries, the majority of them from uncultured bacteria, but also from some isolated strains such as from *Faecalicatena contorta* [*Eubacterium contortum*]. Its high relative abundance strongly suggested that this phylotype was the primary SQ degrader. Contributing 19.5% of the total reads was an ASV affiliated with the genus *Anaerospora* within the family Sporomusaceae of Bacillota. Additionally, a small proportion of reads (7.4%) were affiliated to the genus *Lachnoclostridium* of the family Lachnospiraceae.

We also sequenced the metagenome of the consortium (accessible via the JGI-IMG system, Taxon ID: 3300047135), resulting in three metagenome-assembled genomes (MAGs), each with an estimated completeness of 100% and a redundancy of <5%. Furthermore, two complete 16S rRNA genes could be recovered (IMG gene IDs, Ga0499732_170_207_1739 and Ga0499732_174_24_1707). One of the MAGs was classified as belonging to the genus *Faecalicatena* (*Muricomes* in the Genome Taxonomy Data Base; Parks et al., 2022). With its complete 16S rRNA gene sequence, we found through Blastn (Boratyn et al., 2013) *Faecalicatena* sp. DSM22707 with 99.32% identity (Supplementary Table S1) as the closest isolate that is available in a culture collection. We tested whether *Faecalicatena* sp. DSM22707 could grow with SQ, which it could, as described further below. For the other two MAGs, we could not find any close relatives available in culture collections for testing. The second complete 16S rRNA gene as obtained from the Negativicutes MAG, was assigned to *Sporomusaceae* sp. 2C, with 96.09% identity (query coverage 84%, Supplementary Table S2). However, the alignment of this 16S rRNA gene with the *Anaerospora* ASV gene fragment obtained by Illumina sequencing (see above), showed 99% identity. Therefore, we refer to this MAG as *Anaerospora*.

### 3.2 Growth physiology of the consortium and pure culture of *Faecalicatena* sp. DSM22707

**Figure 4:**
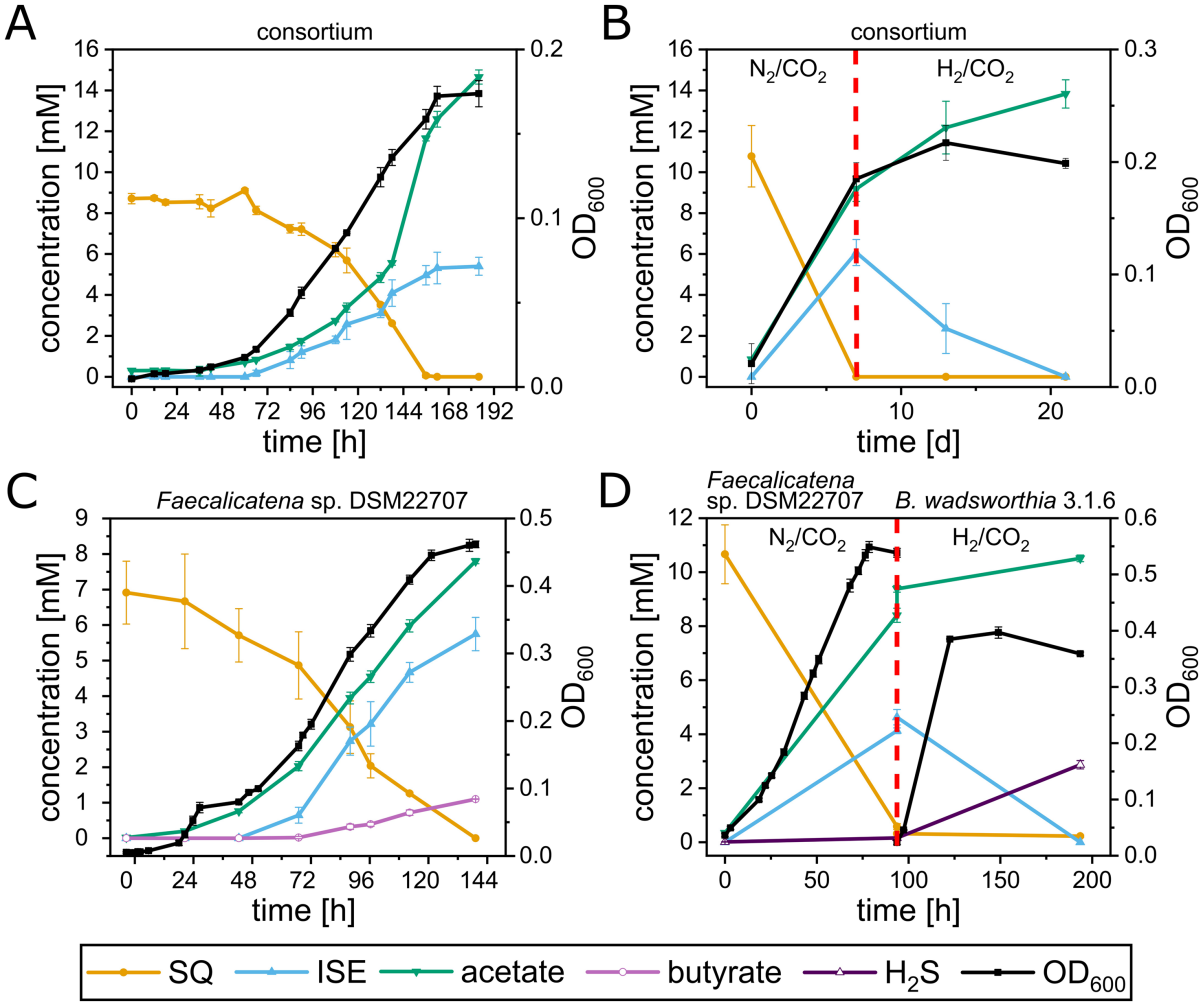
Growth experiments with the highly enriched consortium and with *Faecalicatena* sp. DSM22707 and *B. wadsworthia* 3.1.6 in pure culture, each with SQ as the sole carbon source. **(A)** The consortium completely utilized SQ concomitant with growth and ISE and acetate production; small amounts of DHPS were also detectable (not shown, see text). Furthermore, hydrogen gas (H_2_) was produced (see text and Supplementary Figure S1), which disappeared when the consortia were incubated for additional weeks, while some ISE was degraded (see text and Supplementary Figure S2). **(B)** When the headspace of outgrown consortium-cultures was spiked with additional H_2_ gas after 7 d of incubation (indicated by the red dashed line), the ISE was completely utilized, concomitant with additional acetate production; sulfide was also detected in higher amounts (see text). **(C)** *Faecalicatena* sp. DSM22707 completely utilized SQ with concomitant growth and production of ISE and acetate and produced significant amounts of butyrate. Furthermore, small amounts of SL, SLA and SAA, and succinate and formate were detected, but no H_2_ in the headspace (see text). **(D)** After growth of *Faecalicatena* sp. DSM22707 with SQ, the culture medium was filter-sterilized and inoculated with *B. wadsworthia* 3.1.6 (indicated by the red dashed line); additionally, the headspace was spiked with H_2_ gas. The ISE disappeared completely while additional acetate and sulfide were produced. Each growth experiment was performed at least in triplicate; mean values are given, and error bars correspond to the standard deviation.

The growth of the consortium, and *Faecalicatena* sp. DSM22707 in pure culture, with SQ, was followed in detail, to confirm substrate disappearance and to identify and quantify any products formed. The consortium (Figure 4A) completely degraded SQ (8.7 ± 0.2 mM) within 7.5 d (growth rate μ_max_ = 0.02 h^−1^) concomitant with biomass formation (max. OD_600nm_, 0.17 ± 0.01) and ISE formation (5.4 ± 0.4 mM). In addition to ISE, small amounts of DHPS were detectable when the cultures entered the stationary phase (68 ± 5 μM; not shown in Figure 4A), but no other organosulfonates such as SL, SAc, SLA, 4-deoxy-4-sulfoerythrose or 4-deoxy-4-sulfoerythrulose (see below). Another product released into the culture fluid in high amounts was acetate (14.7 ± 0.3 mM final concentration). When the cultures entered the stationary phase, small amounts of formate (0.97 ± 0.09 mM; not shown in Figure 4A), but no other fermentation products such as butyrate or succinate (see below) were detectable. In the headspace of the cultures, molecular hydrogen (H_2_) production was detected concomitant with growth (up to 3.5 ± 0.1 mM H_2_) (see Supplementary Figure S1). Only small amounts of sulfide (0.23 ± 0.17 mM) were detected in the stationary phase. With continued incubation, we observed that the H_2_ and the ISE concentrations decreased significantly but not completely (see Supplementary Figure S2), and we considered whether the growth of the ISE degrader in the consortium might have been limited by insufficient availability of a suitable electron donor. Therefore, the growth experiment was repeated (Figure 4B) and following the conversion of all SQ to ISE (at day 7 of incubation), we replaced the headspace from N_2_/CO_2_ to H_2_/CO_2_ (80/20%). After an additional incubation of 14 d, no ISE was detected and the sulfide concentration increased (1.6 ± 0.1 mM).

The pure culture of *Faecalicatena* sp. DSM22707 grew with SQ or glucose, each in Ti(III)-reduced freshwater mineral salt medium (Figure 4C), only in the presence of small amounts of yeast extract as a supplement (YE, 0.05% w/v); it therefore exhibited a first, short growth phase with YE (Supplementary Figure S3). Strain DSM22707 degraded SQ (6.9 ± 0.7 mM, *n* = 4) completely within 5 d (μ_max_ = 0.03 h^−1^) concomitant with biomass formation (max. OD_600nm_, 0.47 ± 0.01) and ISE formation (final concentration, 5.8 ± 0.4 mM). No DHPS or SAc was detectable; however, small amounts of SL (up to 0.2 ± 0.024 mM) were detected after growth, as well as trace amounts of SLA and SAA, as identified by high-resolution MS (Supplementary Figures S4, S5); for the latter compounds, we had no standards available for quantification. Other products found to be released into the culture fluid in significant amounts were acetate (final concentration, 7.8 ± 0.1 mM), butyrate (final concentration, 1.1 ± 0.01 mM), and small amounts (not shown in Figure 4C) of succinate (0.16 ± 0.05 mM) and formate (0.13 ± 0.09 mM). No H_2_ was detected in the headspace after growth. For comparison, strain DSM22707 grown with glucose (6.41 ± 0.23 mM) produced acetate (7.87 ± 0.28 mM), butyrate (2.75 ± 0.12 mM), succinate (0.40 ± 0.04 mM), formate (0.48 ± 0.01 mM), but also no H_2_.

In order to reproduce the sulfolytic, two-step of SQ by the consortium (Figure 4B) we used a defined co-culture model. For this, the growth experiment with *Faecalicatena* sp. DSM22707 was repeated (Figure 4D). After SQ was completely utilized by *Faecalicatena* sp. DSM22707 and ISE was produced (at day 4 of incubation), and the culture fluid was filter-sterilized and inoculated with the known ISE-degrading, sulfide-producing isolate *Bilophila wadsworthia* 3.1.6 (Peck et al., 2019; Baron et al., 1989). Additionally, the headspace was switched from N_2_/CO_2_ to H_2_/CO_2_. Strain 3.1.6 completely degraded ISE within 4 d, concomitant with further increases of sulfide (from 0.2 ± 0.01 mM to 2.9 ± 0.2 mM) and acetate (from 8.2 ± 0.1 mM to 10.5 ± 0.1 mM) (Figure 4D).

### 3.3 Candidate gene clusters identified for SQ and ISE degradation in the consortium and SQ degradation in *Faecalicatena* sp. DSM22707

**Figure 5:**
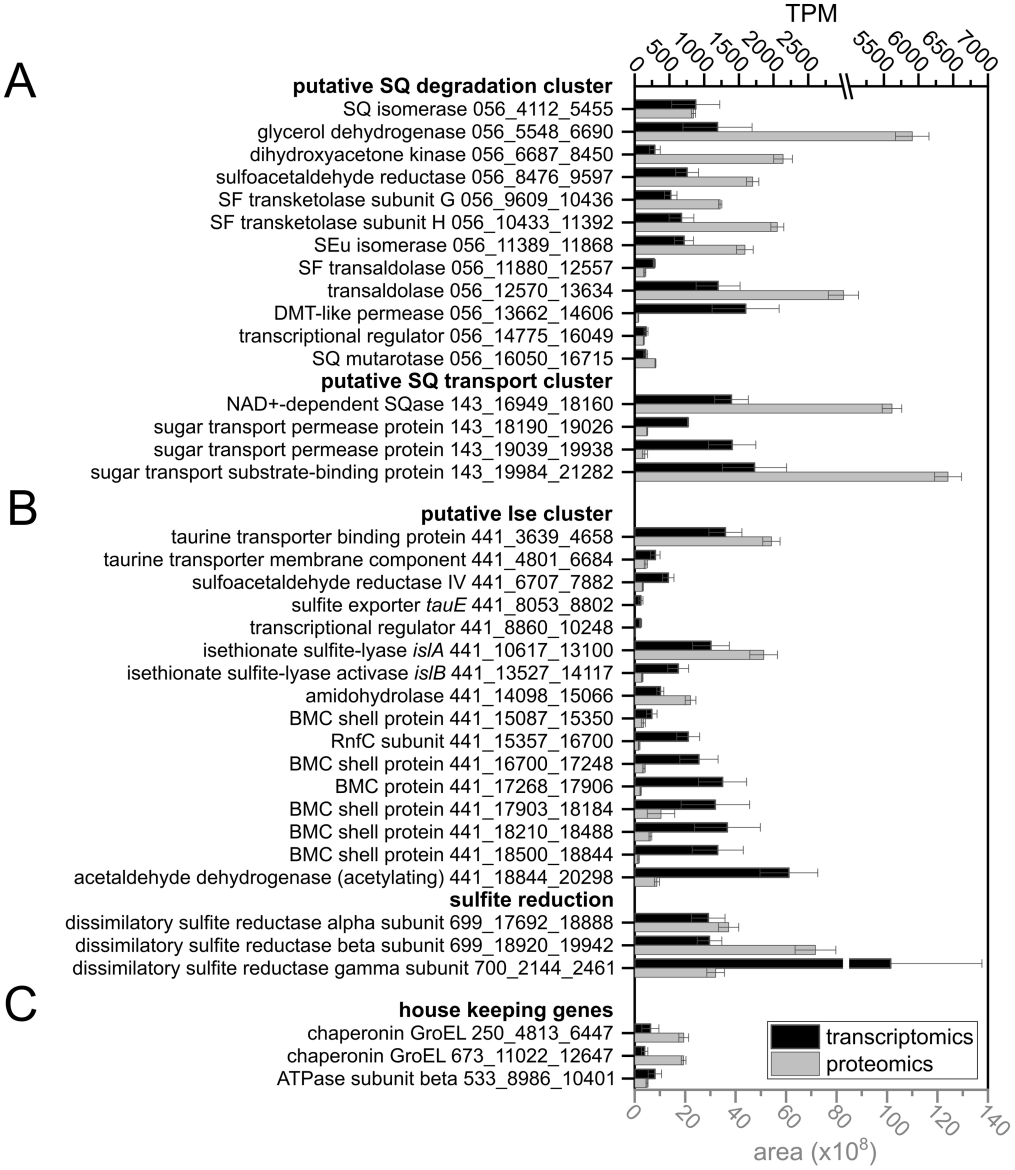
Extract of the data on gene expression levels in the consortium, as determined by transcriptomics and proteomics, showing candidate genes for SQ **(A)** and ISE degradation, including genes for sulfite reduction **(B)** and housekeeping genes **(C)** for comparison. The IMG metagenome annotation was used to build the total-proteomics database and IMG Gene IDs (prefix: Ga0499732_) are indicated as gene identifiers. The gene clusters on scaffolds Ga0499732_056 and _143 for SQ utilization were assigned to the *Faecalicatena* MAG, and scaffolds _441, _699 and _700 for ISE utilization and sulfite respiration were assigned to the *Anaerospora* MAG. Transcript abundance is given in transcripts per million (TPM) and protein abundance is expressed as the mean peak area of all peptides identified by mass spectrometry. Transcriptomic sequencing was performed in duplicate and total proteomics in triplicate when starting with consortium cultures harvested each at late exponential growth phase; error bars correspond to standard deviation. DMT, drug/metabolite transporter; BMC, bacterial microcompartment.

**Figure 6:**
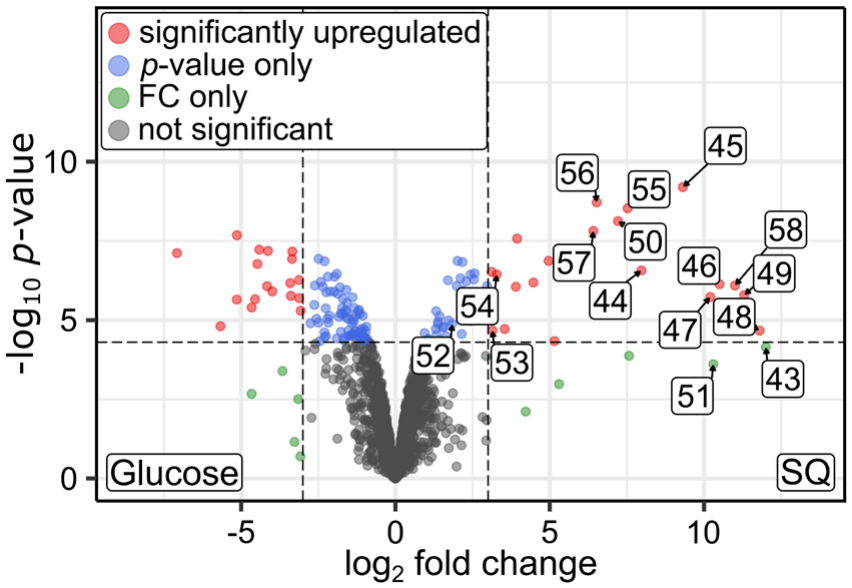
Volcano plot illustrating the differential proteomics results obtained for pure cultures of *Faecalicatena* sp. DSM22707 grown with either 10 mM SQ or glucose. Proteins that were found to be significantly more abundant (up-regulated, as defined by a log_2_ fold change (FC) cutoff of 3 and a *p*-value cutoff of 5×10^−5^) are indicated as red dots, shifted to the right if they were more abundant in SQ-grown cells. In this figure, the proteins encoded by the SQ-metabolism gene cluster are labeled by the last two digits of their IMG Gene IDs (prefix 29568592xx); consecutive numbers indicate that the genes are encoded next to each other.

**Figure 7:**
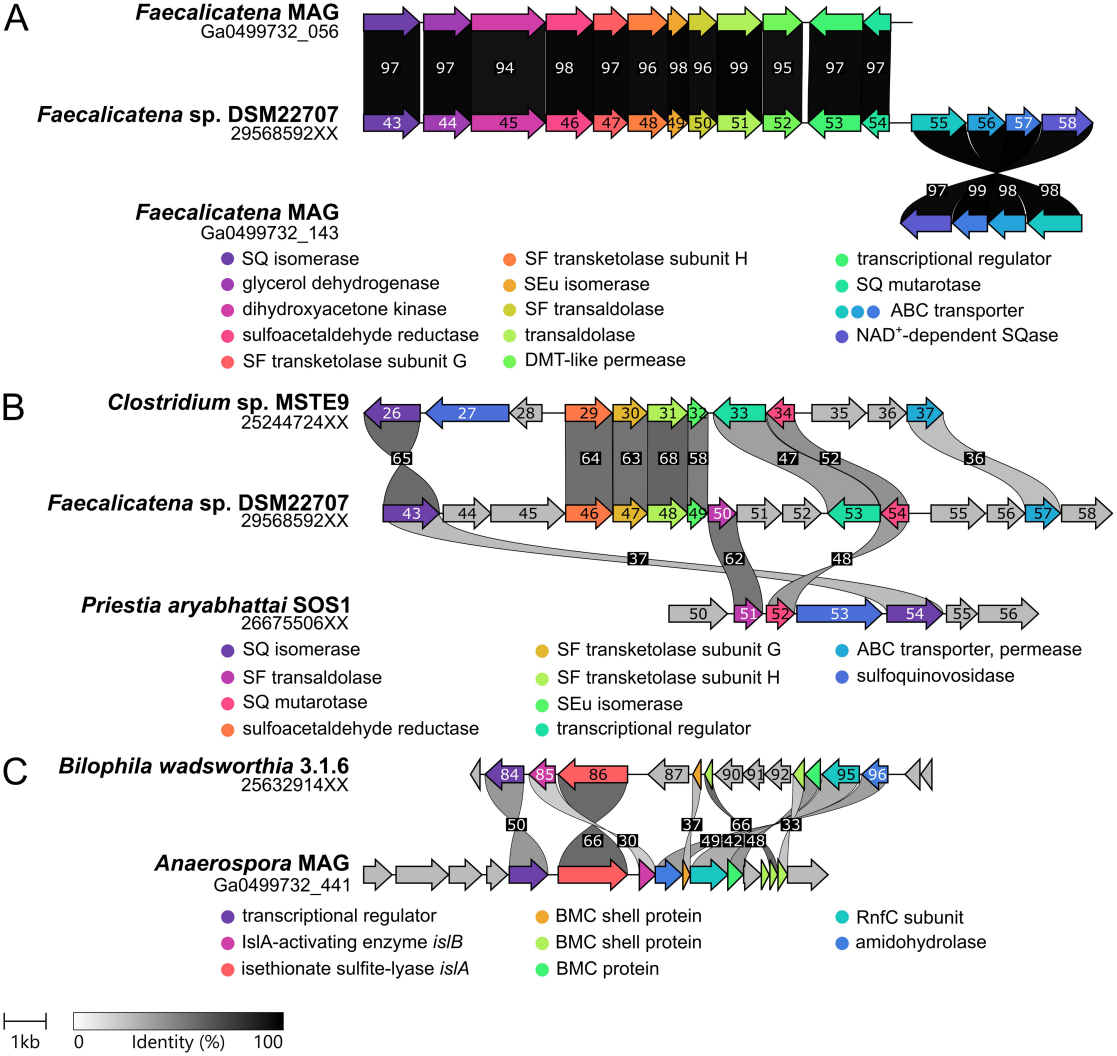
Illustration of the gene clusters for SQ and ISE metabolism as identified by transcriptomics and/or proteomics in this study. **(A)** Comparison of the SQ degradation gene clusters found in the *Faecalicatena*-phylotype of the consortium and in the pure culture of *Faecalicatena* sp. DSM22707. The genes are arranged in the same order and the encoded proteins show ≥94% identical amino acid sequence (id-aa). Note that the predicted SQase and the ABC transporter genes were located on a different scaffold for the consortium metagenome. **(B)** Comparison of the SQ degradation gene cluster of strain DSM22707 with those of *P. aryabhattai* SOS1 (sulfo-TAL pathway) and *Clostridium* sp. MSTE9 (sulfo-TK pathway). Gene 2956859258 was predicted to be NAD^+^-dependent SQase, because of its homology to *Arthrobacter* sp. strain AK01 (see main text). **(C)** Gene cluster identified for ISE desulfonation in the *Anaerospora*-phylotype of the consortium in comparison to the gene cluster of *B. wadsworthia* 3.1.6. IMG gene IDs are indicated for single genome sequences and IMG locus tags (scaffolds) for the consortium metagenome. The digits shown in the gene arrows complete the respective IMG gene IDs (i.e., replace the XX). Sequence identity is indicated in grayscale (numbers, % identity).

In order to examine which enzymes and genes could be employed for SQ and ISE degradation by the consortium and by which phylotype, we analyzed the consortium’s total proteome and transcriptome. All genes that were found to be both transcribed and translated at relevant levels, as compared to housekeeping genes, were screened for homologs of known genes for SQ and ISE degradation. In Figure 5, an excerpt of the data is shown with the identified candidates for SQ metabolism (Figure 5A), encoded by two gene clusters (contigs) each affiliated to *Faecalicatena*, and for ISE metabolism (Figure 5B) encoded by one gene cluster affiliated to *Anaerospora*, compared to representative housekeeping genes (Figure 5C). Furthermore, pure cultures of *Faecalicatena* sp. DSM22707 grown with SQ compared to those grown with glucose were analyzed by differential proteomics of the total proteome. Therefore, its draft genome was sequenced and annotated (accessible via the JGI-IMG system, Taxon ID 2956855026). Figure 6 shows a volcano plot for the differential proteomics dataset, highlighting all genes that were specifically induced during SQ utilization. It identified a gene cluster nearly identical to that of the consortium, comprising the same sets of candidate genes for SQ metabolism in strain DSM22707 (Figure 7A). Interestingly, these clusters included candidate genes for key enzymes of both the sulfo-TK pathway (transketolase, leading to C_2_-sulfonates) and the sulfo-TAL pathway (transaldolase, leading to C_3_-sulfonates) (Figure 7B), as described in the following. Because of the high identity of both gene clusters, in the text below we mostly refer to the IMG gene IDs (295685xxxx) of *Faecalicatena* sp. DSM22707 proteins, and their percent amino-acid sequence identities (% id-aa) relative to genes of the archetype sulfo-TK pathway of *Clostridium* sp. MSTE9 (Liu et al., 2021) and/or sulfo-TAL pathway of *P. aryabhattai* SOS1 (Frommeyer et al., 2020). For the corresponding IMG locus tags in the metagenome, see Figures 5, 7.

For liberation of the SQ headgroup from SQ glycoconjugates, a sulfoquinovosidase (SQase) candidate (IMG gene ID 2956859258) with 35% identity to the recently described NAD^+^-dependent sulfoquinovosidase of *Arthrobacter* sp. strain AK01 (Kaur et al., 2023) was found to be induced in *Faecalicatena* sp. DSM22707 during SQ growth (Figure 6). Such an SQase candidate was also detected by transcriptomics and proteomics in the *Faecalicatena*-phylotype of the consortium (Figure 5A). These respective SQase genes were encoded next to candidate SQ ABC transport genes for *Faecalicatena* sp. DSM22707 (Figure 7A) and the consortium. Furthermore, a candidate SQ mutarotase for catalyzing the anomerization between SQ anomers (Liu et al., 2021; Frommeyer et al., 2020) was produced from both *Faecalicatena* gene clusters (Figures 5A, 6, 7A), for strain DSM22707 (2956859254), which showed52 and 48% id-aa to SQ mutarotase candidates of the sulfo-TK pathway of *Clostridium* sp. MSTE9 (2524472434) and the sulfo-TAL pathway in *P. aryabhattai* SOS1 (2667550652), respectively (Figure 7B); these candidates showed no significant homology to the SQ mutarotase enzyme of *Herbaspirillum seropedicaea* (Abayakoon et al., 2018). Furthermore, SQ isomerase (2956859243) candidates, which showed 65 and 32% id-aa to the SQ isomerases of strains MSTE9 (2524472426) and SOS1 (2667550654) (Figure 7B), were produced (Figures 5A, 6) from each gene cluster, for conversion of SQ to SF. Further downstream in the clusters, two candidate genes each for transketolase subunits were expressed (Figures 5A, 6). The first subunit (2956859247) showed 63% id-aa to the SF transketolase subunit G of strain MSTE9 (2524472430), containing a thiamine diphosphate binding domain (Lindqvist et al., 1992; Nikkola et al., 1994), and the second (2956859248) showed 68% id-aa to the SF transketolase subunit H of strain MSTE9 (2524472431), containing the C-terminal domain with proposed regulatory activity (Nikkola et al., 1994) (Figure 7B). The gene (2956859249) directly downstream was also expressed (Figure 6), encoding a candidate 4-deoxy-4-sulfoerythrulose (SEu) isomerase, with 58% id-aa to the archetype enzyme of the sulfo-TK pathway (2524472432). Interestingly, also two different transaldolase candidate genes further downstream (Figure 7) were produced (Figure 5A), in strain DSM22707 specifically during growth with SQ but not with glucose (Figure 6). The first (2956859250) showed high identity (62% id-aa) to the archetype SF transaldolase enzyme of the sulfo-TAL pathway of *P. aryabhattai* SOS1 (2667550651), while the sequence identity for the second (2956859251) was lower (32% id-aa, 19% query cover) and its protein-family (pfam) entry was fructose-6-phosphate aldolase (see also below).

For the conversion of the SAA (sulfoacetaldehyde) produced by the SF transketolase reactions, candidate SAA reductases were encoded in both *Faecalicatena* gene clusters, of which that of strain DSM22707 (2956859246) was SQ-inducible produced (Figure 6) and showed 64% id-aa to that of *Clostridium* MSTE9 (2524472429). Candidate genes for SLA (3-sulfolactaldehyde) reductases and SLA dehydrogenases were found elsewhere in the consortium metagenome (Supplementary Table S3) or the *Faecalicatena* sp. DSM22707 genome (Supplementary Table S4), for the conversion of the SLA to DHPS or SL, respectively. Three candidates for SLA oxidation or reduction were found to be produced by the consortium as follows: one SLA dehydrogenase candidate with 31% id-aa to that of *P. putida* SQ1 (Felux et al., 2015) in the *Faecalicatena*-phylotype and two SLA reductase candidate in both the *Anaerospora*-phylotype with 37% id-aa and another one in the *Faecalicatena*-phylotype with 30% id-aa to *E. coli* (Denger et al., 2014). In the pure culture of strain DSM22707, candidates for both reduction and oxidation of SLA were produced: for SLA reduction, an annotated 2-hydroxy-3-oxopropionate reductase (2956855439) with the highest id-aa (35%) to the SLA reductase of *E. coli* (Denger et al., 2014), although it was produced only at very low levels and not differentially. Other proteins encoded in the clusters that were also detected to be SQ-inducible produced (Figure 6) are candidates for glycerol dehydrogenase (2956859244) and dihydroxyacetone kinase (2956859245).

Other genes in the clusters encoded transporters, that might facilitate the export of the organosulfonate intermediates, i.e., a drug/metabolite permease (2956859252), or the import of SQ, i.e., a predicted ABC-type transporter (2956859255–2956859257), for which the substrate binding protein (2956859255) was confirmed to be produced during growth with SQ (Figure 6). The substrate binding protein (2956859255) showed 32% id-aa to the characterized SQ binding protein of the ABC transporter of *Agrobacterium tumefaciens* (Gene Bank: AAK90108.1) (Snow et al., 2022). The permease protein (2956859257) showed 37% id-aa to the corresponding protein of *Rhizobium leguminosarum* (Li et al., 2020) and 36% id-aa to that of *Clostridium* sp. MSTE9 (2524472437). Furthermore, the repressor candidate produced (2956859253) (Figure 6) showed 47% id-aa to that of *Clostridium* sp. MSTE9 (2956859233).

For the degradation of ISE, an *Anaerospora*-phylotype gene cluster was identified due to its high expression (Figure 5B) and high homology to the ISE-utilization gene cluster known for *B. wadsworthia* (Figure 7C). The gene cluster encoded the candidate isethionate sulfite lyase (IslA) and its corresponding activating subunit (IslB), which showed high identity (66 and 48% id-aa, respectively) to those of *B. wadsworthia* 3.1.6 (Supplementary Table S5). Additionally, this gene cluster encodes for SAA reductase and CoA-acylating acetaldehyde dehydrogenase (Supplementary Table S5), such as in *B. wadsworthia* (Figure 7C), and these genes were also expressed (Figure 5C). Further proteins encoded in the gene cluster (Figure 7C) and expressed during growth of the consortium with SQ (Figure 5C), are candidate transporters for ISE uptake and/or sulfite/sulfonate export (Supplementary Table S5), and ethanolamine utilization (Eut) proteins EutN, EutQ and EutM. The Eut proteins are components for bacterial microcompartments (BMCs) which likely harbor the IslAB and other enzymes, as described for *B. wadsworthia* (Burrichter et al., 2021). The dissimilatory sulfite reductase (Dsr) complex alpha and beta and DsrC genes, which were found to be highly expressed (Figure 5C) were also affiliated with the *Anaerospora-*phylotype (Figure 7C and Supplementary Table S5).

### 3.4 *In silico* analysis of the SF transketolase and transaldolases

**Figure 8:**
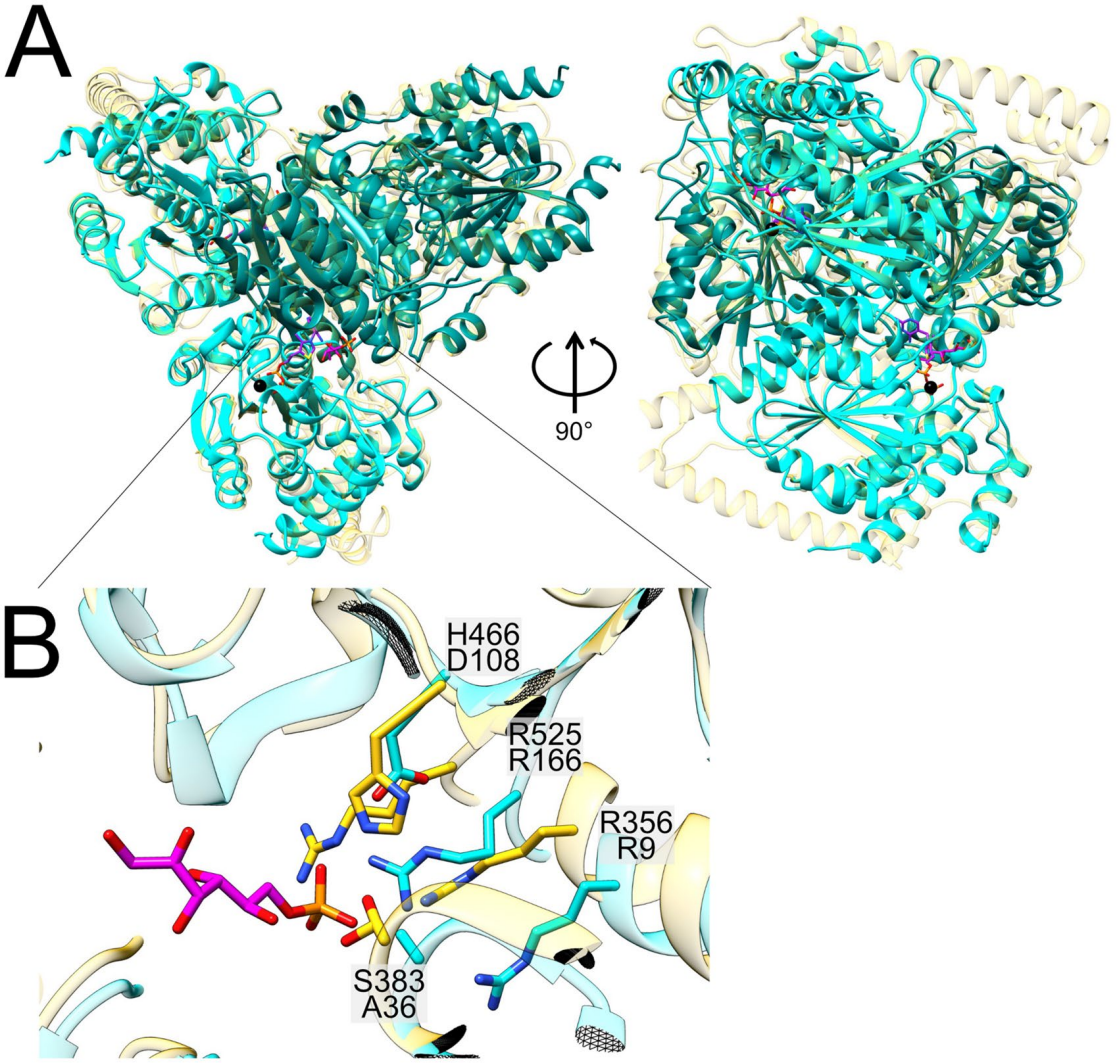
Structural superimposition of the Alphafold-predicted heterotetramer of *Faecalicatena* sp. DSM22707 SF transketolase (N-terminal in teal, C-terminal in cyan) with the crystal structure of the single-gene fructose 6-phosphate transketolase from *Scheffersomyces stipitis* (PDB code: 5XU2; depicted in yellow) containing the bound cofactor thiamine pyrophosphate (TPP, purple), a calcium ion (Ca^2+^, black) and the sugar-phosphate substrate of *S. stipitis* transketolase (F6P, pink) **(A)**. A close-up of the superimposed catalytic centers is also shown **(B)**, in which the differing amino-acid residues are depicted [coloring according to the global structure in **(A)**]. For *Faecalicatena* transketolase, the residue H466 is exchanged for D108, and S383 is exchanged for A36. Residues R525/166 and R356/9 are conserved between the two enzymes **(B)**.

AlphaFold was used to predict (Jumper et al., 2021) the structure of the two subunits G and H of the putative split gene candidate SF transketolase of *Faecalicatena* sp. DSM22707 (2956859247 and 9248, respectively), in multimer mode, to form an ⍺_2_β_2_ heterotetramer, which resembles structurally and biochemically split gene transketolase of *Carboxydothermus hydrogenoformans* (PDBcode: 6yak) (Supplementary Figure S6). A comparison at the residue level was performed against the single-gene transketolase of *Scheffersomyces stipitis* (PDBcode: 5XU2), as this transketolase contains all the necessary cofactors (see below). In addition, the SF transketolase model of Liu et al. (2021) was included for structural comparison (Supplementary Table S6). The residues were numbered according to full-length transketolase (5XU2). As expected, the predicted model revealed a similar domain arrangement to the two crystal structures compared to (6YAK and 5XU2), with two active sites within the ⍺_2_β_2_ heterotetramer (Figure 8).

Transketolases are thiamine pyrophosphate (TPP) and calcium (Ca^2+^) dependent enzymes, and the *Faecalicatena* transketolase ⍺_2_β_2_ heterotetramer has all the necessary residues to bind these cofactors (Figure 8). The calcium ion of 5XU2 is coordinated by the lateral groups of aspartate and asparagine and the main chain of the isoleucine. The residues are strictly conserved in the transketolase subunit G domain (2956859247) of *Faecalicatena* sp. DSM22707 (residues D155, N185 and I187). The TPP binding site is located at the dimer interface in 5XU2, where it forms a curved cavity that results in a high-energy, V-conformation state of the bound TPP (James et al., 2020; Leeper et al., 2005). The identical TPP coordinating residues can be found in the heterotetramere studied here indicating the same TPP binding capabilities. The difference between the enzymes lies in the sugar head group orienting residues, whereas the *S. stipites* and *C. hydrogenoformans* transketolases employ an RSFHR motif for the orientation of the phosphate moiety, while the corresponding SF transketolase subunits harbor an RAFDR motif. This alteration most likely leads to a different substrate specificity (Payongsri et al., 2012, 2015).

**Figure 9:**
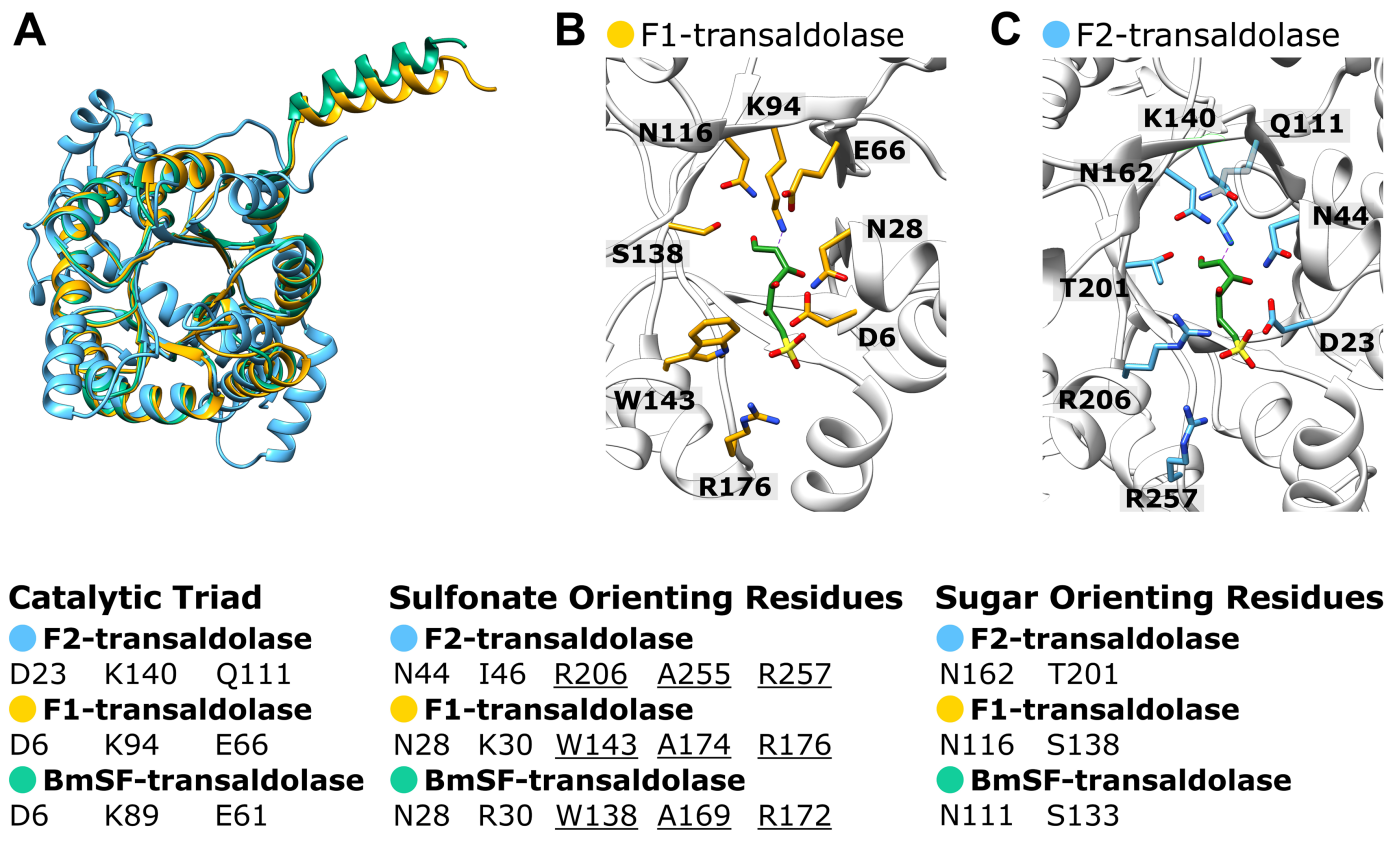
Superimposition of the Alphafold predicted structures of the two *Faecalicatena* sp. DSM22707 transaldolase candidates expressed during SQ utilization (F1, IMG Gene ID: 2956859250; F2, 2956859251) with the cryo-EM structure of *P. megaterium* SF transaldolase (BmSF-TAL, PDB code: 8 BC4) including the SF ligand of BmSF-TAL **(A)**. Also shown are the superimposed catalytic sites of transaldolase candidates F1 and F2 with the BmSF ligand (ligand QC9) as seen in the cryo-EM structure **(B)**. For comparison, the catalytic triad, sulfonate-orienting, and sugar-orienting residues are shown as predicted by Snow et al. (2023), showing that transaldolase F1 contains the same residues as the BmSF enzyme but that the transaldolase F2 differs in its residues **(C)**. Transaldolase BmSF-TAL, green; *Faecalicatena* transaldolase candidates F1 and F2, yellow and blue, respectively.

The AlphaFold structure predictions of the two different transaldolase candidates (Figure 9, Supplementary Figure S6) produced from the *Faecalicatena* sp. DSM22707 gene cluster (see above) were compared to the recently published cryo-EM structure of the SF transaldolase of *P. megaterium* (BmSF-TAL), which organizes into a double ring-like structure of a dimer-of-pentamers holo decamere (Snow et al., 2023). In Figure 9, the first encoded *Faecalicatena* transaldolase candidate (2956859250) is labeled F1, and the second candidate (2956859251) is labeled F2. As each subunit folds into a TIM barrel that harbors an active site, the comparison was only made at the monomer level to reveal potentially similar/differing catalytic capabilities. The postulated sulfonate-orienting WAR motif of BmSF-TAL was indicated to be present also in the first candidate F1 but appeared to be different in the second one, F2 (Figure 9). For transaldolase F1, we found the same residues as in BmSF-TAL for the catalytic triad (D6, K94, E66) and the sugar-orienting residues (N166, S138), while the catalytic triad in the second transaldolase candidate F2 (D23, K140, Q111) and its sugar-orienting residues (N162, T201) are different compared from BmSF-TAL (D6, K89, E61 and N111, S133, respectively).

## 4 Discussion

Approximately 10 years ago, the first bacterial pathway and genetic markers for key enzymes in SQ degradation were discovered, initiating a still ongoing discovery of an intriguing array of alternative bacterial SQ degradation pathways. At present, these can be categorized into five different biochemical strategies (see Introduction) for breaking down and directing SQ carbon into central metabolism, each of which drives the growth of a wide range of heterotrophic bacteria in diverse habitats, from aerobic to anaerobic, including the human gut microbiome (Hanson et al., 2021). For the glycolytic SQ pathways, several pathway variants have been identified to date, with different strategies to further utilize the remaining C_3_ or C_2_ sulfono-aldehyde cleavage intermediate in bacterial metabolism, either as an internal electron acceptor in SQ-fermenting bacteria (excreting DHPS or ISE) (Burrichter et al., 2018; Liu et al., 2021; Denger et al., 2014; Frommeyer et al., 2020), or as an additional electron donor for electron-transport driven ATP generation in respiring bacteria (excreting SL) (Felux et al., 2015; Frommeyer et al., 2020), and, if energetically feasible, by coupling its oxidation also directly to substrate-level phosphorylation for additional ATP generation, excreting SAc (Chu et al., 2023) or 3-sulfopropanoate (Chen et al., 2024).

**Figure 10:**
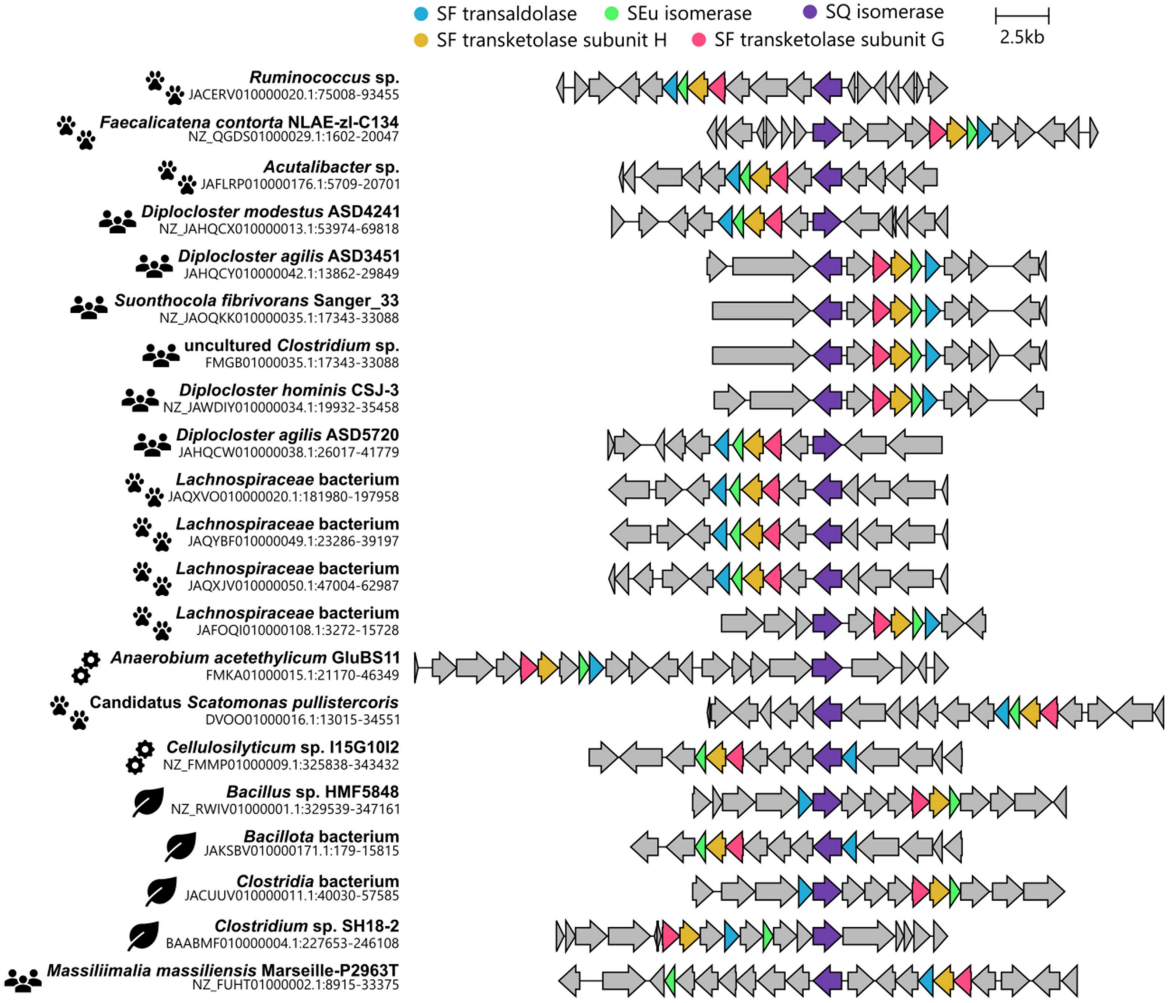
Illustration of gene clusters found in genomes of cultured bacteria (isolates) or MAGs of uncultured bacteria, each highlighted for a co-localization of valid candidate genes for SF transaldolases, SF transketolases, and SQ and SEu isomerases. The pictograms indicate the origin of the isolate/MAG which are animal feces or intestinal contents (paw prints), human feces (humans), engineered environments (cogwheels), or natural environments (leaves). For further details on their origin, see Supplementary Table S7.

In this study, we have uncovered another pathway variation, that is, a bifurcated SQ transaldolase/transketolase pathway in strictly anaerobic members of the family Lachnospiraceae. The family Lachnospiraceae is one of the most abundant taxa in the human gut and rumen microbiota and can be associated with health as well as various intra- and extraintestinal diseases (e.g., Meehan and Beiko, 2014; Vacca et al., 2020). The architecture of the identified gene cluster with *co*-localized (candidate) SF transaldolase and SF transketolase genes, each associated with genes for SQ and SEu isomerases, was found to be predominantly represented (15 out of 21) in bacterial isolates or MAGs obtained from human or animal intestinal contents or feces (cow, pig, chicken, pika), or for example from a biogas reactor, marine sediment or a pond of a mud volcano, as shown in Figure 10 (further details on their origin can be found in Supplementary Table S7). From the taxonomic distribution of this bifurcated SQ-pathway gene cluster, it is tempting to speculate that the evolutionary context for the development and selection of such a pathway may involve substrate-rich and strictly anaerobic (highly reduced) habitat conditions. Notably, the initial enrichment culture, the now defined consortium (Figures 2, 3), which we have kept viable for almost a decade, originated from strictly anoxic Lake Constance sediment, specifically from the FeS-rich layer representing the eutrophic/mesotrophic period of the lake (Ibrahim et al., 2021). Furthermore, we used a highly reduced culture medium (see Materials and methods). Now, a genome-sequenced bacterial isolate employing this pathway variant is available for further experimental examination in pure culture, *Faecalicatena* sp. DSM22707, and in defined mixed culture, e.g., with *Bilophila wadsworthia*, as demonstrated in this study (Figure 4).

**Figure 11:**
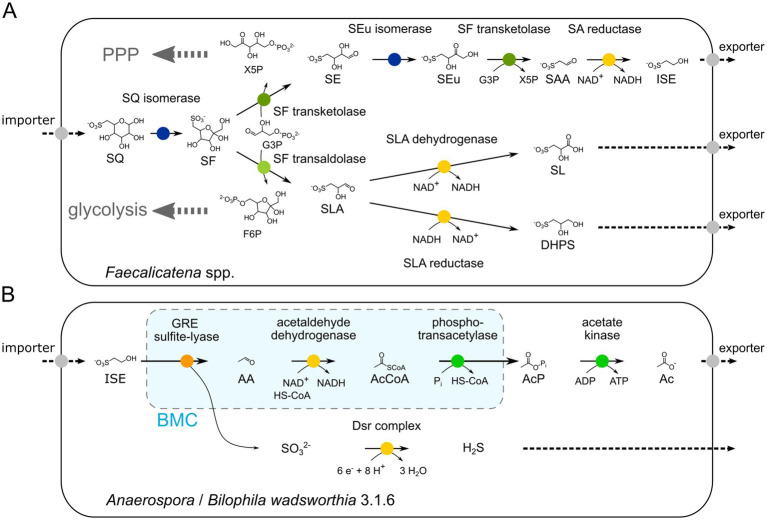
Illustration of the proposed bifurcated sulfo-TK and sulfo-TAL pathway in the *Faecalicatena* species **(A)** and the desulfonation pathway for the major excreted product, ISE, in the *Anaerospora* phylotype of the consortium and *B. wadsworthia* 3.1.6 of the defined co-culture **(B)**, as examined in this study. The growth physiology experiments showed that SQ is degraded predominantly to ISE, but that SLA and SL (pure culture) or DHPS (consortium) are also produced and excreted in small amounts, providing a strong indication that the pathway bifurcates at the cleavage of the C_6_-backbone of SQ to yield both C_2_- and C_3_ intermediates. Corresponding SF transketolase and SF transaldolase candidate enzymes were found to be encoded in a gene cluster that was transcribed and/or translated along with valid SF isomerase candidates for the upstream pathway, and SEu isomerase and SAA and SLA reductase and dehydrogenase candidates for the pathways downstream of the bifurcation. The generated F6P and X5P entry into either glycolysis or pentose phosphate pathway (PPP) is also indicated **(A)**. The ISE is utilized for organosulfonate respiration by the *Anaerospora* phylotype or *Bilophila wadsworthia* 3.1.6 when employing the GRE sulfite-lyase reaction in bacterial microcompartments (BMC) (light blue), as indicated by the transcriptional/proteomics data and as previously described (Peck et al., 2019; Burrichter et al., 2021) **(B)**. AA, acetaldehyde, AcCoA, acetyl-CoA, AcP, acetyl phosphate, Ac, acetate, Dsr, dissimilatory sulfite reductase.

While a demonstration of the predicted enzyme activities and an *in vitro* reconstitution of the proposed pathway by recombinant enzymes was beyond the scope of this study, the results of the growth physiology experiments (Figure 4), the transcriptomic and/or (differential) proteomic examinations of the consortium and pure culture (Figures 5, 6), and the high sequence homologies of the identified candidate enzymes with previously confirmed enzymes of other SQ pathways (Figures 7A,B), strongly suggest that this novel pathway most likely proceeds as shown in Figure 11. The growth physiology experiments showed that SQ is predominantly degraded to ISE, but that SLA and SL (pure culture) or DHPS (consortium) are also produced and excreted in small amounts under the cultivation conditions used in this study. The products formed are key indicators that the pathway bifurcates at the cleavage of the C_6_-backbone of SQ to yield both C_2_- and C_3_-intermediates. Corresponding candidate enzymes, SF transketolase, and SF transaldolase, were found to be encoded in the gene cluster and to be highly transcribed and/or translated during growth with SQ, along with valid candidates for SF isomerases for funneling the SQ into the pathway, and for candidate SEu isomerase, and SAA and SLA reductase and dehydrogenases for the two pathway branches downstream of the bifurcation.

The attribution of the implied SF transketolase activity to the two identified subunits encoded in *Faecalicatena* is plausible because of their high sequence identity to the characterized enzyme subunits of *Clostridium* sp. MSTE9 (Figure 7) and their high production in strain DSM22707 specifically during growth with SQ (Figure 6). In addition, the structural model of the predicted α_2_β_2_ heterotetramer of *Faecalicatena* sp. DSM22707 (Figure 8) and the sequence conservation at the substrate and cofactor binding sites indicate that the enzyme can catalyze the proposed reaction. Furthermore, the sugar-orienting residues are strictly conserved between the reference transketolases and the *Faecalicatena* SF transketolase candidate, and the reaction mechanism of the cleavage of the non-sulfonated C_2_ unit can be expected to function in the same way as described by Leeper et al. (2005). The difference in the accepted substrates may be explained by differences in the head group orienting residues (see Results). The observation that mutation of these residues in transketolases can lead to the acceptance of different substrates (Payongsri et al., 2012, 2015) supports the idea that these changes make it possible to accept sulfonate groups compared to phosphate esters.

The attribution of the SF transaldolase activity to the identified candidate encoded by *Faecalicatena* is plausible because of its high sequence identity to the characterized enzymes from *P. aryabhattai* and *B. megaterium* (Figure 7), and its high production in strain DSM22707, particularly during growth with SQ (Figure 6). Furthermore, the structural prediction is consistent with the cryo-EM structure of *B. megaterium* SF transaldolase (BmSF-TAL) (Figure 9), and the catalytic residues and the orientation of the sugar moiety proposed by Snow et al. (2023) are conserved. This suggests that the transfer of the non-sulfonated C_3_ unit of SF to the acceptor molecule occurs via the same Schiff-base mediated reaction. The second transaldolase candidate identified shows variation in the key residues (see Results), questioning its ability to perform the same reaction. The observed substitutions shift the chemical potential of the respective residues from acid to amide (E61Q) in the catalytic triad, and from hydrophobic to basic (W138R) in the sulfonate-orienting residues, while the substitution (S133T) in the sugar orienting residues does not necessarily affect the enzyme capabilities. These observations support the idea that the enzyme, as annotated, may resemble an F6P (fructose 6-phosphate) aldolase, rather than an SF transaldolase, thus cleaving F6P to dihydroxyacetone and G3P (glyceraldehyde-3-phosphate) (Schürmann and Sprenger, 2001). When considering such a putative aldol cleavage of the F6P as produced in the SF transaldolase branch of the pathway (Figure 11), it is tempting to speculate whether the additional G3P generated may feed ‘across’ the bifurcation to support the SF transketolase branch of the pathway with its higher carbon yield (C_1_-C_4_) than in the SF transaldolase branch (C_1_-C_3_), but involving two G3P-dependent reactions and, thus, higher demand in G3P (Figure 11). In this context, *Faecalicatena* sp. DSM22707 is also likely to be able to utilize also sulfoquinovosyl glycerol (SQG) as a substrate, as a highly induced candidate gene for an NAD^+^-dependent sulfoquinovosidase (Kaur et al., 2023) was detected specifically during growth with SQ (Figure 6), for the generation of glycerol and SQ. In addition, SQ-induced candidates for glycerol dehydrogenase and dihydroxyacetone kinase were detected (Figure 6), which may help to funnel both the glycerol from SQG and also the dihydroxyacetone from the putative F6P-aldol cleavage mentioned above into the pathway as additional G3P.

The results of the growth physiology experiments with the consortium and with strain DSM22707 also extend the known non-sulfonated fermentation products that can be formed during SQ fermentation, for example in the human gut, ranging from acetate, succinate, and formate, as observed for *E. coli* mixed acid SQ fermentation (Burrichter et al., 2018), to also butyrate, as observed for the SQ-fermenting DSM22707 culture, or molecular hydrogen (H_2_), as identified for the consortium in this study, and as tentatively identified previously for pure cultures of *C. symbiosum* and *E. rectale* (Frommeyer et al., 2020). Strain DSM22707 grown with glucose produced the same range of fermentation products except ISE, with a higher relative amount of butyrate. For the consortium, the range of fermentation products observed was smaller, most likely because any butyrate and succinate formed (as well as most of the H_2_) may have been utilized by the *Anaerospora* member as a carbon and/or electron source during organosulfonate respiration with ISE. These results shed further light on the complexity of the different possibilities of cross-feeding of the SQ-carbon and electrons, and of the sulfonate intermediate as electron donor and/or acceptor (see below), through the anaerobic microbial food chain and across completely different types of metabolism: From the SQ-fermenting microbe, further to commensal organosulfonate-respiring microbes, and especially in the context of the gut microbiome, and with the produced short-chain fatty acids (SCFAs) acetate and butyrate, probably also to the host with all its implications (Lee et al., 2022; Christl et al., 1996; Niekamp and Kim, 2023). This adds to the biomedical relevance of the hydrogen sulfide (H_2_S) produced in the human gut during the complete degradation of SQ via DHPS (Hanson et al., 2021; Liu et al., 2020) and possibly also via ISE (Wei et al., 2022), SL (Frommeyer et al., 2020), and/or SAc (Chu et al., 2023).

For the consortium, the ISE produced was most likely utilized by the second most prominent phylotype, *Anaerospora*. A gene cluster from this MAG, highly similar to the ISE-utilization gene cluster from *Bilophila wadsworthia* involving the glycyl-radical enzyme (GRE) isethionate sulfite-lyase (Figure 11) (Peck et al., 2019; Xing et al., 2019), was found to be highly expressed and produced, but none of the other four known bacterial pathways for isethionate desulfonation (Morimoto et al., 2024). Therefore, this organism is most likely utilizing the ISE as a sulfite donor for energy generation through sulfite respiration, driven by an external electron donor. The GRE reaction also appears to be catalyzed within a bacterial microcompartment (BMC) in *Anaerospora* (Figure 11), as homologs of the *Bilophila* BMC shell proteins and the RnfC-like subunit were highly represented in the transcriptomic and proteomic data (Figure 5B) (Burrichter et al., 2021; Doron and Kerfeld, 2024).

The growth experiments with the consortium and with the defined co-culture model of strain DSM22707 and *B. wadsworthia*, further demonstrated that a quantitative utilization of the amount of ISE produced by the SQ fermentation as sulfite donor and electron acceptor by the second-tier bacterium (*Anaerospora* or *Bilophila*) can only be achieved, if additional amounts of (external) electron donor, in this case, H_2_, are provided (Figures 4B,D). This suggests that in these two experimental systems, insufficient amounts of appropriate electron donors are produced and excreted by the SQ-fermenting organism, be it H_2_ gas or other fermentation products, which can be used as electron donors by the ISE-respiring bacteria. In the anoxic environments and the gut microbiome, such additional electron donors can be derived from other fermentations and may be acquired in competition with, for example, methanogens and sulfate reducers. Furthermore, these observations are in contrast to the results of growth experiments with *E. coli* and *Desulfovibrio* sp. (Burrichter et al., 2018), in which *E. coli* produced DHPS from SQ as a fermentation product and *Desulfovibrio* quantitatively degraded the DHPS via desulfonation of SL, that is, *Desulfovibrio* used the DHPS both as an energy and carbon source (via pyruvate) and as an electron acceptor (via sulfite) without the need for additional electron donors other than the DHPS from SQ. The contrasting observations reflect the higher number of carbon atoms abstracted from SQ and/or the higher oxidation state of the sulfonate intermediates excreted, that is, from SQ to the C_2_-sulfonates ISE or SAc, rather than to the C_3_-sulfonates DHPS or SL. The ISE may be further oxidized to SAc, and the DHPS and SL may be oxidized via pyruvate, and each is employed for ATP production via substrate-level phosphorylation. Intriguingly, these metabolic options are also mirrored in two of the SQ pathway variants, where the SAA is oxidized to SAc rather than reduced to ISE (Chu et al., 2023) (Figure 1), while a similar pathway has most recently been demonstrated to exist from DHPS to 3-sulfopropanoate (not shown; see Chen et al., 2024), each for additional ATP generation.

The intriguing diversity of SQ utilization options revealed to date in bacteria certainly reflects the interconnection of overall pathway energetics with the cost of investing in different numbers and amounts of enzymes (proteins) to catalyze these reactions efficiently, and the interconnection of aerobic or anaerobic lifestyles with the overall electron and carbon balances within a bacterium, as well as in between the different bacteria when dealing with more complex environments. When trying to understand the specific advantages or disadvantages of the many SQ metabolic options, as demonstrated once again in this study, and when trying to explain their evolutionary fine-tuning, one must also consider the types of partner organisms available in the respective habitats, in addition to the type of organosulfonate intermediate that is actually transferred (DHPS, SL, ISE, SAc, or even 3-sulfopropanoate). In addition, what co-substrates may be accessible, such as non-sulfonated sugars or SQ-glycerol that may be fed into the transaldolase/transketolase reactions, or the presence or absence of additional electron donors, such as H_2_ that may support desulfonation by second-tier members. One might expect that the discovery phase for SQ metabolic options in bacteria is reaching a plateau (although one should always expect the unexpected), but their enzymatic and energetic details, their activity in different environmental habitats or gut microbiome settings, and especially their eco-physiological context of evolutionary fine-tuning, harbor many more, very interesting questions.

## Data Availability

The metagenome of the consortium and the draft genome of Faecalicatena sp. DSM22707 can be accessed via IMG/JGI (https://img.jgi.doe.gov/) using the IMG Taxon Object IDs 3300047135 and 2956855026, respectively. Raw reads of the Illumina amplicon sequencing and transcriptomics of the consortium are accessible from NCBI (https://www.ncbi.nlm.nih.gov/) with the BioSample accession SAMN43364791 and BioProject accession PRJNA1152503, respectively. Raw data of the proteomics can be accessed from ProteomeXchange (https://www.proteomexchange.org/) via the identifier PXD055296.

## Footnotes

^1^https://inkscape.org

^2^https://www.biologie.uni-konstanz.de/proteomics-centre/

^3^https://www.cgl.ucsf.edu/chimera
