# Supplementary material for: Anaerobic Faecalicatena spp. degrade sulfoquinovose via a bifurcated 6-deoxy-6-sulfofructose transketolase/transaldolase pathway to both C2- and C3-sulfonate intermediates
Source: Front Microbiol. 2024 Dec 5;15:1491101. doi: 10.3389/fmicb.2024.1491101 (PMC11659671; doi:10.3389/fmicb.2024.1491101)
Supplement: Supplementary file 1 [file Data_Sheet_1.pdf]

Supplementary Material

Anaerobic *Faecalicatena* spp. degrade sulfoquinovose *via* a bifurcated 6-deoxy-6-sulfofructose transketolase/transaldolase pathway to both C_2- and C_3-sulfonate intermediates

Sabrina Borusak[1,2,3]*, Karin Denger[1,4], Till Dorendorf[1,2], Corentin Fournier[1,3], Harry Lerner[1,3], Olga Mayans[1,2], Dieter Spiteller[1,2], David Schleheck[1,2,3]*

[1]Department of Biology, University of Konstanz, Konstanz, Germany
[2]The Konstanz Research School Chemical Biology, University of Konstanz, Konstanz, Germany
[3]Limnological Institute, Department of Biology, University of Konstanz, Konstanz, Germany
*Shared corresponding authorship: Sabrina Borusak, sabrina.borusak@uni-konstanz.de and David Schleheck, david.schleheck@uni-konstanz.de

Table of contents

Supplementary Table S1 2
Supplementary Table S2 3
Supplementary Table S3 4
Supplementary Table S4 5
Supplementary Table S5 7
Supplementary Table S6 9
Supplementary Table S7 11
Supplementary Figure S1 13
Supplementary Figure S2 14
Supplementary Figure S3 15
Supplementary Figure S4 16
Supplementary Figure S5 17
Supplementary Figure S5 18
Supplementary References 19

Supplementary Table S1 Top 10 NCBI blastn alignment results for the 16*S* rRNA gene with the IMG gene ID Ga0499732_170_207_1739 from the consortium excluding uncultured bacteria and models. The strain which is *Faecalicatena* sp. DSM22707 is indicated in bold.

Description	Scientific Name	Max Score	Total Score	Query Cover	E value	Per. ident	Acc. Len	Accession
Eubacterium contortum strain Ala-1 16*S* ribosomal RNA gene, partial sequence	*Faecalicatena contorta*	2761	2761	99%	0	99.47	1520	EU980608.1
Muricomes sp. strain DSM 1596-AFMT-0%NaCl-C04 16*S* ribosomal RNA gene, partial sequence	*Muricomes* sp.	2724	2724	97%	0	99.66	1505	OQ607135.1
***Eubacteriaceae* bacterium PEH A 16*S* ribosomal RNA gene, partial sequence**	***Eubacteriaceae* bacterium PEH A**	**2667**	**2667**	**95%**	**0**	**99.32**	**1471**	**GQ920790.1**
Bacterium 14(2016) 16*S* ribosomal RNA gene, partial sequence	bacterium 14(2016)	2638	2638	94%	0	99.38	1455	KT889289.1
Faecalicatena contorta strain DSM 3982 16*S* ribosomal RNA, partial sequence	*Faecalicatena contorta*	2617	2617	99%	0	97.7	1524	NR_117147.1
Faecalicatena fissicatena strain DSM 3598 16*S* ribosomal RNA, partial sequence	*Faecalicatena fissicatena*	2617	2617	99%	0	97.7	1522	NR_104800.1
Faecalicatena contorta strain DSM 3982 16*S* ribosomal RNA, partial sequence	*Faecalicatena contorta*	2612	2612	99%	0	97.7	1521	NR_104803.1
Faecalicatena fissicatena 16*S* ribosomal RNA, partial sequence	*Faecalicatena fissicatena*	2584	2584	99%	0	97.25	1526	NR_117563.1
Faecalicatena fissicatena strain DSM 3598 16*S* ribosomal RNA, partial sequence	*Faecalicatena fissicatena*	2582	2582	99%	0	97.24	1524	NR_117140.1
Eubacterium sp. SB2 partial 16*S* rRNA gene, strain SB2	*Eubacterium* sp. SB2	2575	2575	97%	0	97.66	1506	LN850732.1

Supplementary Table S2 Top 10 NCBI blastn alignment results for the 16*S* rRNA gene with the IMG gene ID Ga0499732_174_24_1707 from the consortium including uncultured bacteria and models.

**Description**	**Scientific Name**	**Max Score**	**Total Score**	**Query Cover**	**E value**	**Per. ident**	**Acc. Len**	**Accession**
Uncultured Clostridia bacterium clone IRB3 16*S* ribosomal RNA gene, partial sequence	uncultured Clostridia bacterium	2375	2465	88%	0	96.31	1525	DQ069197.1
Uncultured Clostridia bacterium clone FW29 16*S* ribosomal RNA gene, partial sequence	uncultured Clostridia bacterium	2368	2484	88%	0	96.45	1518	DQ069215.1
Sporomusaceae bacterium 2C_5 16*S* ribosomal RNA gene, partial sequence	*Sporomusaceae* bacterium 2C_5	2331	2331	84%	0	96.09	1535	OR541989.1
Sporomusaceae bacterium 2C_4 16*S* ribosomal RNA gene, partial sequence	*Sporomusaceae* bacterium 2C_4	2331	2331	84%	0	96.09	1457	OR541988.1
Sporomusaceae bacterium 2C_2 16*S* ribosomal RNA gene, partial sequence	*Sporomusaceae* bacterium 2C_2	2331	2331	85%	0	95.97	1511	OR541986.1
Uncultured bacterium clone G07 16*S* ribosomal RNA gene, partial sequence	uncultured bacterium	2322	2432	90%	0	95.32	1557	AF407698.1
Uncultured *Veillonellaceae* bacterium clone NJFU_SLX-S177 16*S* ribosomal RNA gene, partial sequence	uncultured *Veillonellaceae* bacterium	2259	2369	88%	0	95.19	1513	KJ128018.1
Sporomusaceae bacterium 2C_3 16*S* ribosomal RNA gene, partial sequence	*Sporomusaceae* bacterium 2C_3	2252	2252	82%	0	95.78	1397	OR541987.1
Uncultured bacterium gene for 16*S* ribosomal RNA, partial sequence, clone: TSNSR001_I02	uncultured bacterium	2224	2224	86%	0	94.2	1539	AB487544.1
Uncultured bacterium clone LAC3 16*S* ribosomal RNA gene, partial sequence	uncultured bacterium	2215	2215	97%	0	91.16	1636	JQ004085.1

Supplementary Table S3 NCBI blastp alignment results of proteins from the consortium to SLA dehydrogenase (DH) of *Pseudomonas (Ps) putida* SQ1 (1) and *Priestia (P) aryabhattai* SOS1 (2) and SLA reductase of *E. coli* (3) and *Lachnoclostridium (L.) symbiosum* (2); the reference (ref.) organism for which the best hit was found is indicated. The MAG in which the candidate enzyme was found given below the IMG Gene ID (Prefix: Ga0499732_). Mean areas of proteomics data (*n* = 3) and mean transcripts per million (TPM; *n* = 2) with standard deviation are indicated. Only hits with an Identity above 30% are shown.

IMG Gene ID (MAG)	IMG Annotation	Identity	Query Cover	e-value	ref. organism	Proteomics	TPM
**SLA dehydrogenase**							
118_6469_7977 (*Hungatella*)	glyceraldehyde-3-phosphate dehydrogenase (NADP+)	37.06%	100%	2.00E-105	*P. aryabhattai*	not detected	0.3 ± 0.1
633_9444_10931 (*Hungatella*)	acyl-CoA reductase-like NAD-dependent aldehyde dehydrogenase	35.27%	96%	2.00E-96	*Ps. putida*	not detected	0.2 ± 0.0
068_8247_9794 (Anaerospora)	1-pyrroline-5-carboxylate dehydrogenase	33.33%	92%	9.00E-66	*Ps. putida*	not detected	2.0 ± 0.7
570_14443_15948 (*Faecalicatena*)	acyl-CoA reductase-like NAD-dependent aldehyde dehydrogenase	32.69%	84%	2.00E-67	*P. aryabhattai*	not detected	0.6 ± 0.2
469_17655_19205 (Faecalicatena)	1-pyrroline-5-carboxylate dehydrogenase	31.15%	91%	5.00E-71	*Ps. putida*	5.E+08 ± 4.E+07	58.3 ± 19.5
379_19064_20578 (*Hungatella*)	acyl-CoA reductase-like NAD-dependent aldehyde dehydrogenase	30.34%	29%	3.00E-06	*P. aryabhattai*	not detected	not detected
**SLA reductase**							
703_20685_21341 (*Anaerospora*)	trk system potassium uptake protein TrkA	41.03%	13%	0.007	*E. coli*	not detected	9.1 ± 3.0
618_16613_17497 (*Anaerospora*)	2-hydroxy-3-oxopropionate reductase	37.37%	93%	2.00E-45	*E. coli*	1.E+09 ± 2.E+08	50.3 ± 8.0
039_20068_20727 (*Anaerospora*)	trk system potassium uptake protein TrkA	36.84%	12%	0.019	*E. coli*	not detected	97.1 ± 30.7
081_6584_7468 (*Faecalicatena*)	2-hydroxy-3-oxopropionate reductase	35.71%	93%	5.00E-47	*E. coli*	not detected	0.9 ± 0.3
449_1593_2501 (*Anaerospora*)	3-hydroxyisobutyrate dehydrogenase-like beta-hydroxyacid dehydrogenase	32.16%	93%	4.00E-35	*E. coli*	not detected	0.2 ± 0.1
660_12397_13287 (*Faecalicatena*)	2-hydroxy-3-oxopropionate reductase	30.85%	98%	1.00E-35	*E. coli*	not detected	0.2 ± 0.1
035_15347_16207 (*Faecalicatena*)	3-hydroxyisobutyrate dehydrogenase/2-hydroxy-3-oxopropionate reductase	30.32%	92%	4.00E-33	*E. coli*	7.E+08 ± 3.E+07	78.5 ± 28.3

Supplementary Table S4 NCBI blastp alignment results of proteins from *Faecalicatena* sp. DSM22707 to SLA dehydrogenase (DH) of *Pseudomonas (Ps) putida* SQ1 (1) and *Priestia (P) aryabhattai* SOS1 (2) and SLA reductase of *E. coli* (3) and *Lachnoclostridium (L.) symbiosum* (2); the reference (ref.) organism for which the best hit was found is indicated. The IMG Gene ID (Prefix: 295685) is given. Mean areas of proteomics data (*n* = 3) for glucose (Gluc) and SQ grown cells, with standard deviation, are indicated, as well as the ratio of SQ vs Gluc with p-value, as calculated with the DEP package (see materials and methods)

**IMG Gene ID**	**IMG Annotation**	**Identity**	**Query Cover**	**e-value**	**ref. organism**	**Proteomics**		
						**SQ vs Gluc ratio (*p*-value)**	**mean LFQ.intensity (Gluc)**	**mean LFQ.intensity (SQ)**
**SLA reductase**								
5439	2-hydroxy-3-oxopropionate reductase	35.00%	93%	1.00E-46	*E. coli*	-1.15 (0.0605192)	9.83E+06 ± 5.93E+06	3.49E+06 ± 4.79E+05
7491	glycerol-3-phosphate dehydrogenase (NAD(P)+)	34.88%	14%	0.005	*E. coli*	-0.0907 (0.4245916)	1.31E+07 ± 1.18E+06	1.14E+07 ± 8.30E+05
8941	3-hydroxyisobutyrate dehydrogenase-like beta-hydroxyacid dehydrogenase	32.04%	98%	3.00E-38	*L. symbiosum*	-1.68 (3.78E-05)	1.63E+07 ± 3.17E+06	4.60E+06± 2.21E+05
8944	2-hydroxy-3-oxopropionate reductase	31.80%	98%	3.00E-41	*L. symbiosum*	-1.62 (7.95E-06)	1.63E+07 ± 1.46E+06	4.90E+06 ± 4.30E+05
8998	2-hydroxy-3-oxopropionate reductase	30.59%	88%	9.00E-27	*L. symbiosum*	-0.445 (0.0064904)	4.72E+06 ± 6.21E+05	3.18E+06 ± 1.34E+05
8381	3-hydroxyisobutyrate dehydrogenase	29.09%	92%	3.00E-32	*E. coli*	0.186 (0.0549865)	5.39E+07 ± 2.54E+06	5.64E+07 ± 1.78E+06
9611	6-phosphogluconate dehydrogenase	25.41%	60%	4.00E-05	*L. symbiosum*	-0.543 (0.001241)	1.13E+07 ± 5.31E+05	7.16E+06 ± 5.21E+05
7142	3-hydroxybutyryl-CoA dehydrogenase	25.00%	30%	0.01	*E. coli*	not detected		
7026	6-phosphogluconate dehydrogenase	22.57%	71%	6.00E-05	*E. coli*	not detected		

Supplementary Table S4 continued

IMG Gene ID	IMG Annotation	Identity	Query Cover	e-value	ref. organism	Proteomics		
						SQ vs Gluc ratio (*p*-value)	mean LFQ.intensity (Gluc)	mean LFQ.intensity (SQ)
**SLA dehydrogenase**								
5372	acyl-CoA reductase-like NAD-dependent aldehyde dehydrogenase	32.20%	84%	2.00E-68	*P. aryabhattai*	not detected		
5135	propionaldehyde dehydrogenase	31.14%	33%	2.00E-11	*P. aryabhattai*	not detected		
6413	1-pyrroline-5-carboxylate dehydrogenase	31.09%	91%	6.00E-71	*Ps. putida*	0.0484 (0.6867157)	1.10E+08 ± 1.38E+07	1.05E+08 ± 6.24E+06
6995	propionaldehyde dehydrogenase	29.76%	33%	3.00E-08	*P. aryabhattai*	-1.37 (0.0019919)	2.19E+06 ± 6.25E+05	not detected
5935	malonate-semialdehyde dehydrogenase (acetylating)/methylmalonate-semialdehyde dehydrogenase	29.40%	96%	1.00E-57	*P. aryabhattai*	-0.393 (0.0319108)	3.39E+06 ± 5.26E+05	2.36E+06 ± 1.32E+05
8854	aldehyde dehydrogenase (NAD+)	28.26%	83%	2.00E-37	*P. aryabhattai*	not detected		
7989	malonate-semialdehyde dehydrogenase (acetylating)/methylmalonate-semialdehyde dehydrogenase	28.16%	96%	1.00E-51	*P. aryabhattai*	-0.983 (3.55E-05)	3.30E+07 ± 1.64E+06	1.54E+07 ± 8.57E+05
9445	succinate-semialdehyde dehydrogenase	25.89%	66%	6.00E-10	*P. aryabhattai*	7.57 (0.000134)	2.35E+05 ± 1.67E+05	5.20E+07 ± 3.70E+06
5960	acetaldehyde dehydrogenase/alcohol dehydrogenase	25.28%	33%	1.00E-08	*Ps. putida*	-1.03 (0.0039811)	3.32E+06 ± 8.30E+05	1.46E+06 ± 1.84E+05
7687	acetaldehyde dehydrogenase/alcohol dehydrogenase	23.49%	63%	1.00E-09	*P. aryabhattai*	1.7 (5.53E-07)	1.01E+07 ± 5.39E+05	3.02E+07 ± 1.04E+06
6963	succinate-semialdehyde dehydrogenase	21.88%	63%	4.00E-05	*P. aryabhattai*	0.49 (0.0013635)	5.47E+08 ± 4.34E+07	7.07E+08 ± 3.10E+07

Supplementary Table S5 NCBI blastp alignment results of proteins from the consortium to enzymes described for organosulfonate degradation. Only enzymes with significant hits are shown. TRAP = tripartite ATP-independent periplasmic

IMG Locus Tag Ga0499732_	IMG Annotation	Homologue / IMG Gene ID (Organism)	%Identity	Query cover [%]	e-value	Reference
056_4112_5455	L-fucose-isomerase-like protein	SQ isomerase SqvD / 2524472426 (*Clostridium* sp. MSTE9)	65.77	99	0.0	(4)
056_5548_6690	glycerol dehydrogenase	NADH-dependent sulfoacetaldehyde reductase SqwF/ 2524472429 (*Clostridium* sp MSTE9)	24.43	51	3e-04	(4)
056_8476_9597	alcohol dehydrogenase	NADH-dependent sulfoacetaldehyde reductase SqwF/ 2524472429 (*Clostridium* sp MSTE9)	64.61	100	0.0	(4)
056_9609_10436	transketoalse	6-deoxy-6-sulfofructose transketolase SqwG/ 2524472430 (*Clostridium* sp MSTE9)	64.53	69	8e-133	(4)
056_10433_11392	transketolase	6-deoxy-6-sulfofructose transketolase SqwH / 2524472431 (*Clostridium* sp MSTE9)	70.74	97	6e-160	(4)
056_11389_11868	ribose-5-phosphate isomerase	4-deoxy-4-sulfoerythrulose isomerase SqwI / 2524472432 (*Clostridium* sp MSTE9)	59.24	98	6e-70	(4)
056_11880_12557	transaldolase	6-deoxy-6-sulfofructose transaldolase SqvA / 646729461 (*Bacillus megaterium* DSM1804)	62.83	100	4e-104	(5)
056_12570_13634	transaldolase	6-deoxy-6-sulfofructose transaldolase SqvA / 646729461 (*Bacillus megaterium* DSM1804)	31.88	19	4e-07	(5)
056_14775_16049	Predicted NBD HSP70 family sugar kinase	putative transcriptional regulator / 2524472433 (*Clostridium* sp MSTE9)	48.77	95	1e-146	(4)
056_16050_16715	Hypothetical protein	putative SQ mutarotase SqvB / 2524472434 (*Clostridium* sp MSTE9)	51.58	100	2e-83	(4)
143_18190_19026	Multiple sugar transport system permease protein	ABC transporter, permease protein / 2524472437 (*Clostridium* sp MSTE9)	37.02	94	1e-51	(4)

Supplementary Table S5 continued

IMG Locus Tag Ga0499732_	IMG Annotation	Homologue / IMG Gene ID (Organism)	%Identity	Query cover [%]	e-value	Reference
143_19984_21282	Multiple sugar transport system substrate-binding protein	ABC transporter, solute-binding protein / 2524472435 (*Clostridium* sp MSTE9)	26.67	41	1e-07	(4)
441_3639_4658	Tripartite ATP independent transporter DctP family solute receptor	Isethionate-binding periplasmatic protein DctP / 637782146 (*Desulfovibrio alaskensis* G20)	41.85	91	1e-84	(6)
441_4801_6684	C4-dicarboxylate transporter DctM subunit	Isethionate TRAP transporter permease protein DctMQ / 637782145 (*Desulfovibrio alaskensis* G20)	51.45	97	0.0	(6)
441_6707_7882	Alcohol dehydrogenase class IV	Sulfoacetaldehyde reductase SarD/ 2563290519 (*Bilophila wadsworthia* 3.1.6)	31.40	95	1e-51	(6)
441_8053_8802	Uncharacterized membrane protein YfcA	Sulfite/sulfonate exporter SqwE / 2524472428 (*Clostridium* sp MSTE9)	23.63	93	6e-13	(4)
441_10617_13100	Formate-C acetyltransferase	Isethionate sulfite-lyase catalytical subunit IslA / 2563291486 (*Bilophila wadsworthia* 3.1.6)	66.02	100	0.0	(6)
441_13527_14117	Pyruvate formate lyase activating subunit	Isethionate sulfite-lyase activating subunit IslB / 2563291485 (*Bilophila wadsworthia* 3.1.6)	47.96	100	1e-64	(6)
441_18844_20298	Acetaldehyde dehydrogenase (acetylating)	Acetaldehyde dehydrogenase / 2563291487 (*Bilophila wadsworthia* 3.1.6)	29.53	77	2e-45	(6)
699_17692_18888	Sulfite reductase alpha subunit	Dissimilatory sulfite reductase alpha subunit DsrA / 2524893820 (*Desulfovibrio desulfuricans desulfuricans* DSM642)	50.84	97	2e-137	(6)
699_18920_19942	Sulfite reductase beta subunit	Dissimilatory sulfite reductase beta subunit DsrB / 2524893819 (*Desulfovibrio desulfuricans desulfuricans* DSM642)	52.12	99	1e-134	(6)
700_2144_2461	tRNA 2-thiouridine synthesizing protein E	Dissimilatory sulfite reductase, gamma subunit / 637124027 (*Desulfovibrio vulgaris* DSM 644)	55.24	100	9e-45	(7)

Supplementary Table S6 Comparison of orienting residues of the transketolase models. F1: *Faecalicatena* sp. DSM22707 transketolase domain G, IMG Gene ID: 2956859247, F2: *Faecalicatena* sp. DSM22707 transketolase domain H, IMG Gene ID: 2956859248, Cl1: *Clostridium* sp. MSTE9 transketolase domain 1, IMG Gene ID: 2524472430, Cl2: *Clostridium* sp. MSTE9 transketolase domain 2, IMG Gene ID: 2524472431, 5XU2: *Scheffersomyces stipitis* transketolase, 6YAK AAA: *Carboxydothermus hydrogenoformans* transketolase subunit 1, 6YAK BBB: *Carboxydothermus hydrogenoformans* transketolase subunit 2

**Orientation of**	**Enzyme**	**Orienting residues**							
Calcium	F1	D147	N177	M179					
	Cl1	D148	N178	M180					
	5XU2	D155	N185	I187					
	6YAK AAA	D148	N178	L180					
TPP	F1	G32	K67	H69	GSL116-118	GDGE146-149	N-M-I177-181	K239	H253
	Cl1	G33	K69	H70	GSL117-119	GDGE147-150	N-M-I178-182	K240	H254
	5XU2	A30	N65	H66	GPL114-116	GDGC154-157	N-I-I185-189	I248	H261
	6YAK AAA	G33	K68	H70	GSL117-119	GDGE147-150	N-L-I178-182	K240	H254
	F2	V55	L33	E57	F82	H120			
	Cl2	V61	L39	E63	F88	H126			
	5XU2	V413	L380	E415	F442	H478			
	6YAK BBB	I53	L33	E55	F80	H188			

Supplementary Table S6 continued

**Orientation of**	**Enzyme**	**Orienting residues**				
Fructose	F1	H29	H102	I181	H253	
	Cl1	H30	H103	I182	H254	
	5XU2	H27	H100	I189	H261	
	6YAK AAA	H30	H103	I182	H254	
	F2	L33	F79	N116		
	Cl2	L39	F85	N122		
	5XU2	L380	F439	D474		
	6YAK BBB	L31	F77	D114		
Phosphate	F2	R9	A36	F79	D108	R166
	Cl2	R15	A42	F85	D114	R172
	5XU2	R356	S383	F439	H466	R525
	6YAK BBB	R7	S34	F77	H106	R164

Supplementary Table S7. Available information on the origin and, if appropriate, hosts of bacterial isolates or MAGs that were found to harbor candidate transaldolase/transketolase pathway gene clusters (see Figure 11 in main text).

Organism	NCBI Reference Sequence	Isolation source / Host	MAG/ Isolate	Title in NCBI or IMG, or publication title if available
Ruminococcus sp.	JACERV010000020.1	Cow rumen	MAG	Metabolic interactions driving microbial diversity in a xylose-fermenting chemostat enrichment culture
Faecalicatena contorta NLAE-zl-C134	NZ_QGDS01000029.1	Cow rumen	Isolate	The Hungate 1000. A catalogue of reference genomes from the rumen microbiome (Gold Stamp ID Gp0127393)
Acutalibacter sp.	JAFLRP010000176.1	Intestinal content of plateau pika (*Ochotona curzoniae*)	MAG	Multi-omics revealed taxonomic characterization and physiological potential of uncultured microbiota of plateau pika
Diplocloster modestus ASD4241	NZ_JAHQCX010000013.1	Human feces (healthy children)	Isolate	*Diplocloster agilis* gen. nov., sp. nov. and *Diplocloster modestus* sp. nov., two novel anaerobic fermentative members of *Lachnospiraceae* isolated from human faeces (DOI: 10.1099/ijsem.0.005222)
Diplocloster agilis ASD3451	JAHQCY010000042.1	Human feces (healthy children)	Isolate	*Diplocloster agilis* gen. nov., sp. nov. and *Diplocloster modestus* sp. nov., two novel anaerobic fermentative members of *Lachnospiraceae* isolated from human faeces (DOI: 10.1099/ijsem.0.005222)
Suonthocola fibrivorans Sanger_33	NZ_JAOQKK010000035.1	Human feces	Isolate	Automated analysis of genomic sequences facilitates high-throughput and comprehensive description of bacteria (DOI: 10.1038/s43705-021-00017-z)
uncultured *Clostridium* sp.	FMGB01000035.1	Human feces (healthy individuals)	MAG	Culturing of 'unculturable' human microbiota reveals novel taxa and extensive sporulation (DOI: 10.1038/nature17645)
Diplocloster hominis CSJ-3	NZ_JAWDIY010000034.1	Human feces	Isolate	*Anaerolentibacter hominis* gen. nov. sp. nov., *Diplocloster hominis* sp. nov. and *Pilosibacter fragilis* gen. nov. sp. nov., isolated from human faeces (DOI: 10.1099/ijsem.0.006359)
Diplocloster agilis ASD5720	JAHQCW010000038.1	Human feces (healthy children)	Isolate	*Diplocloster agilis* gen. nov., sp. nov. and *Diplocloster modestus* sp. nov., two novel anaerobic fermentative members of *Lachnospiraceae* isolated from human faeces (DOI: 10.1099/ijsem.0.005222)
Lachnospiraceae bacterium	JAQXVO010000020.1	Pig feces (*Sus scrofa domesticus*)	MAG	Metagenome-assembled genomes from pasture and conventional swine fecal metagenomes

Supplementary Table S7 continued

Organism	NCBI Reference Sequence	Isolation source / Host	MAG/ Isolate	Title in NCBI or IMG, or publication title if available
Lachnospiraceae bacterium	JAQYBF010000049.1	Pig feces (*Sus scrofa domesticus*)	MAG	Metagenome-assembled genomes from pasture and conventional swine fecal metagenomes
Lachnospiraceae bacterium	JAQXJV010000050.1	Pig feces (*Sus scrofa domesticus*)	MAG	Metagenome-assembled genomes from pasture and conventional swine fecal metagenomes
Lachnospiraceae bacterium	JAFOQI010000108.1	Cow rumen	MAG	A highly-resolved spatial and functional map of the ruminant gastrointestinal microbiome
Anaerobium acetethylicum GluBS11	FMKA01000015.1	Biogas slurry	Isolate	*Anaerobium acetethylicum* gen. nov., sp. nov., a strictly anaerobic, gluconate-fermenting bacterium isolated from a methanogenic bioreactor (DOI: 10.1099/ijsem.0.000410
Candidatus *Scatomonas pullistercoris*	DVOO01000016.1	Chicken feces (*Gallus gallus*)	MAG	Extensive microbial diversity within the chicken gut microbiome revealed by metagenomics and culture (DOI: 10.7717/peerj.10941)
Cellulosilyticum sp. I15G10I2	NZ_FMMP01000009.1	Coal seam gas water treatment pond (anaerobic enrichment)	Isolate	Draft genome sequence of *Cellulosilyticum* sp. I15G10I2, a novel bacterium isolated from a coal seam gas water treatment pond (DOI: 10.1128/genomeA.01616-16)
Bacillus sp. HMF5848	NZ_RWIV01000001.1	Solar salt evaporated from seawater	Isolate	*Bacillus* sp. HMF5848 genome sequencing and assembly
Bacillota bacterium	JAKSBV010000171.1	Coral colonies (*Porites lutea* and *Goniastrea edwardsi*)	MAG	Greater functional diversity and redundancy of coral endolithic microbiomes align with lower coral bleaching susceptibility (DOI: 10.1038/s41396-022-01283-y)
Clostridia bacterium	JACUUV010000011.1	Bubbling pool in an active mud volcano	MAG	Sulfur and methane-oxidizing microbial community in a terrestrial mud volcano revealed by metagenomics (DOI: 10.3390/microorganisms8091333)
Clostridium sp. SH18-2	BAABMF010000004.1	Marine sediment	Isolate	*Clostridium sediminicola* sp. nov., a spore-forming anaerobic bacterium isolated from marine sediment
Massiliimalia massiliensis Marseille-P2963T	NZ_FUHT01000002.1	Human feces	Isolate	Description and genomic characterization of *Massiliimalia massiliensis* gen. nov., sp. nov., and *Massiliimalia timonensis* gen. nov., sp. nov., two new members of the family *Ruminococcaceae* isolated from the human gut (DOI: 10.1007/s10482-018-01223-x)

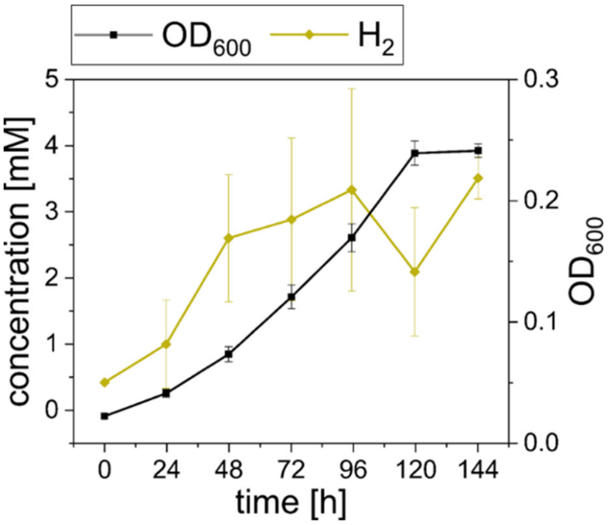

Supplementary Figure S1. Formation of hydrogen gas in the headspace of consortium cultures during growth with SQ (Fig. 4, main text), relative to biomass formation (OD).

Supplementary Figure S2. Extended incubation of the growth experiment with the consortium and SQ as substrate (Fig. 4, main text). When the cultures were incubated for weeks longer after the supplemented 8.4 ± 0.4 mM SQ had been utilized, also approx. 4 mM of the produced ISE was degraded concomitant with a decrease in molecular hydrogen of approx. 2 mM and increase in acetate of approx. 2mM.

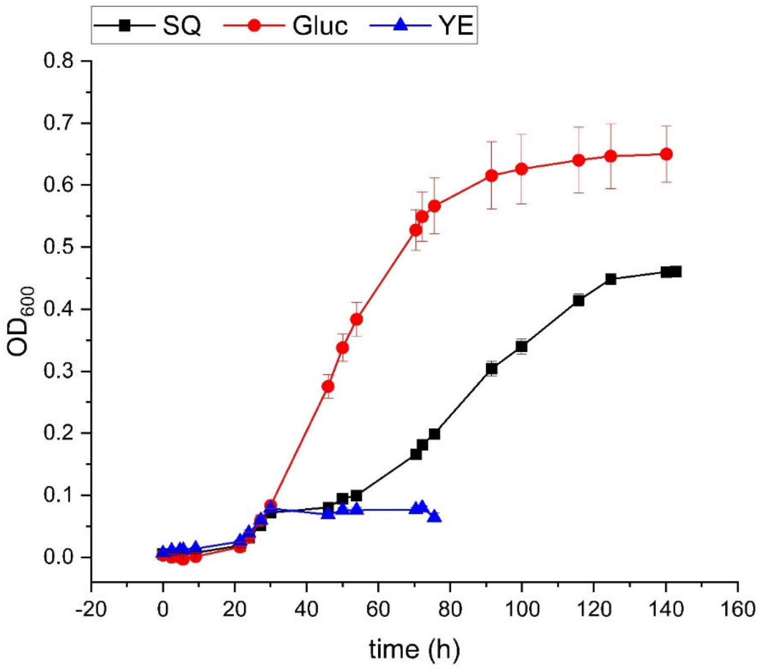

Supplementary Figure S3. Growth experiment with for *Faecalicatena* sp. DSM22707 with either 7 mM SQ (black) or 10 mM glucose (red) each in culture medium supplemented with 0.05% yeast extract (YE), or with 0.05% YE alone (blue). No growth with SQ or glucose was observed in absence of YE as supplement (not shown).

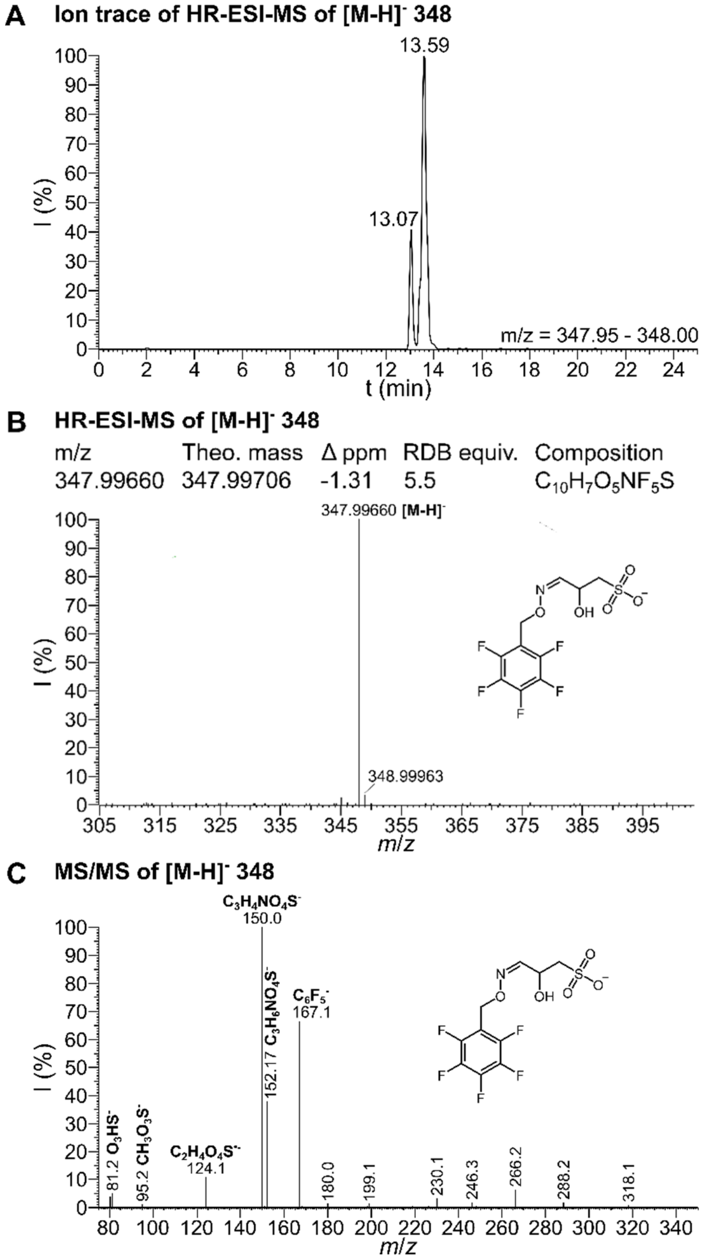

Supplementary Figure S4 Identification of sulfolactaldehyde after derivatization with pentafluorobenzylhydroxylamine (PFBHA) in spent medium of *Faecalicatena* sp. DSM2270 with SQ as sole carbon and energy source. (**A**) Ion trace chromatogram of PFBHA derivatized sulfolactaldehyde with two peaks at 13.1 and 13.6 min, resulting likely from syn- and anti-isomers of PFBHA derivatized sulfolactaldehyde. (**B**) PFBHA derivatized sulfolactaldehyde was detected as $[M-H]^-$ quasimolecular ion at *m/z* 347.99660 matching to the molecular composition $C_{10}H_7O_5NF_5S$ (calculated 347.99706 g/mol, Δ ppm -1.31) as determined by high resolution ESI mass spectrometry. (**C**) The MS/MS spectrum of the quasimolecular ion $[M-H]^-$ at *m/z* 348 revealed specific fragments corresponding to PFBHA derivatized sulfolactaldehyde.

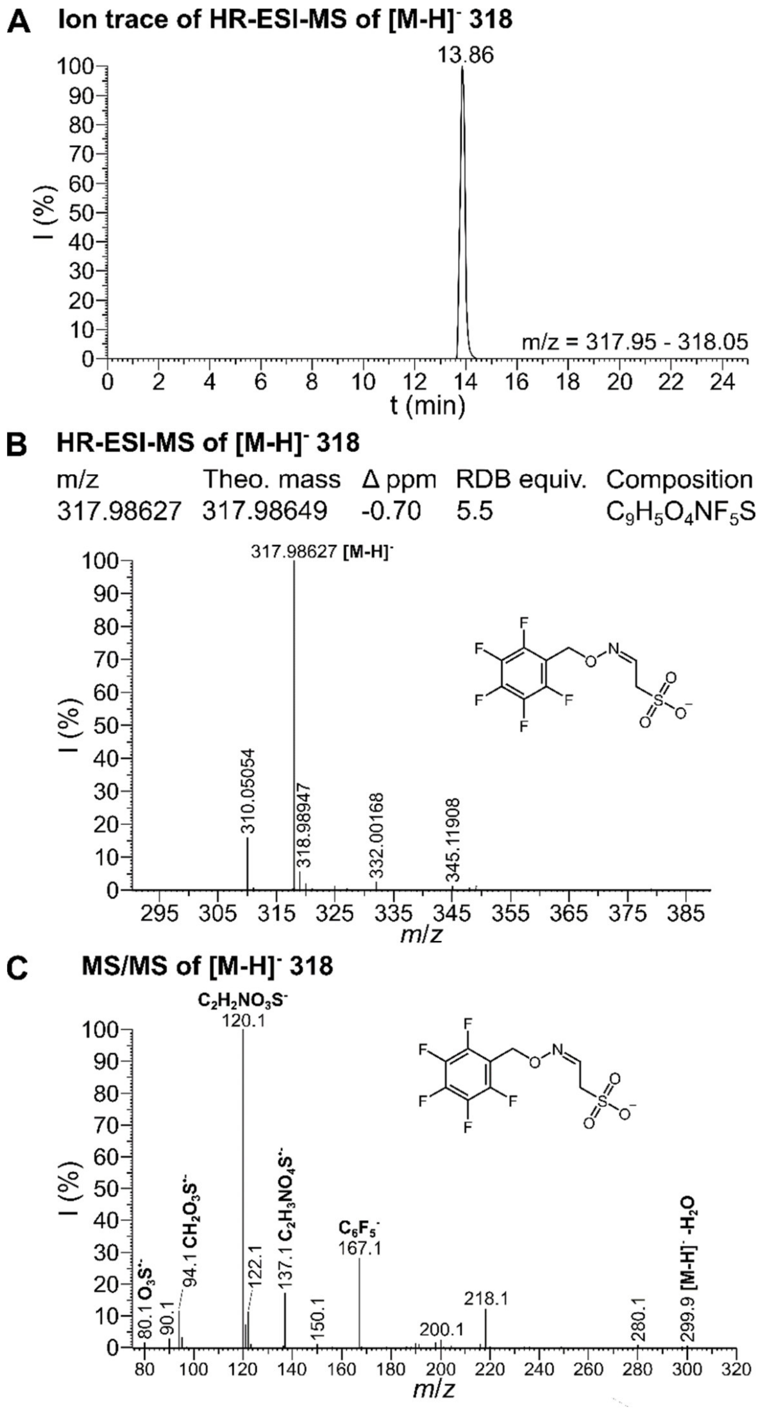

Supplementary Figure S5 Identification of sulfoacetaldehyde after derivatization with pentafluorobenzylhydroxylamine (PFBHA) in spent medium of *Faecalicatena* sp. DSM2270 with SQ as sole carbon and energy source. (**A**) Ion trace chromatogram of PFBHA derivatized sulfoacetaldehyde at 13.9 min. (**B**) PFBHA derivatized sulfoacetaldehyde was detected as $[M\text{-}H]^-$ quasimolecular ion at *m/z* 317.98627 matching to the molecular composition $C_9H_5O_4NF_5S$ (calculated 317.98649 g/mol, Δ ppm -0.70) as determined by high resolution ESI mass spectrometry. (**C**) The MS/MS spectrum of the quasimolecular ion $[M\text{-}H]^-$ at *m/z* 318 revealed specific fragments of PFBHA-derivatized sulfoacetaldehyde.

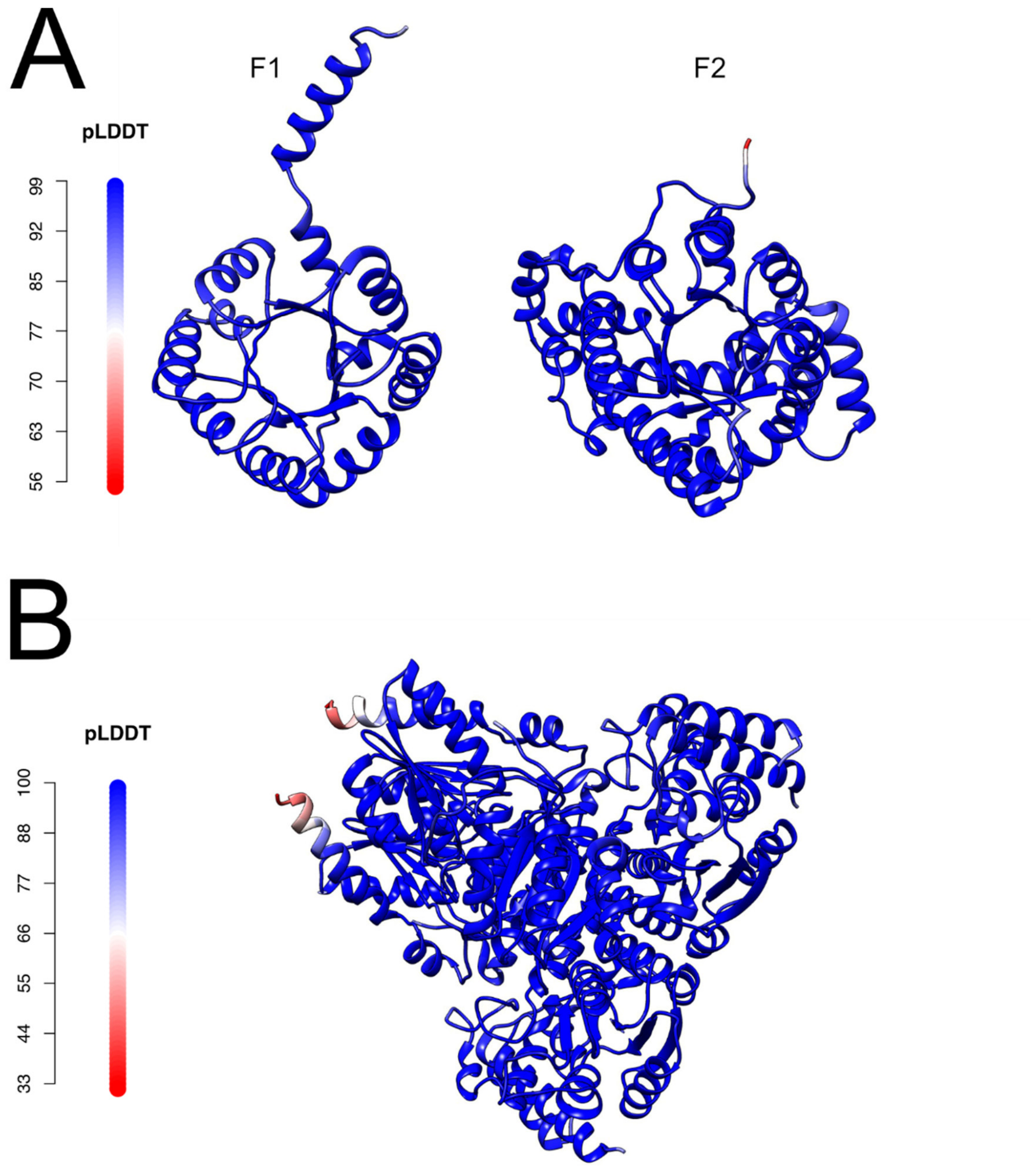

Supplementary Figure S6: AlphaFold prediction accuracy of transaldolases and transketolases. Shown here are the AlphaFold prediction accuracies for the models as indicated by the pLDDT (per-residue Local Distance Difference Test) shown by the scale bar on the left hand side of each enzyme type. Values below 70 are deemed unreliable and potentially unstructured by Alpha Fold. Models calculated for the two *Faecalicatena* sp. DSM22707 transaldolase candidates F1 and F2 separately (**A**). $\alpha_2\beta_2$ heterotetramere of transketolase subunits G and H as predicted in the multimere mode of AlphaFold (**B**).

Supplementary References

1. Felux AK, Spiteller D, Klebensberger J, Schleheck D. Entner-Doudoroff pathway for sulfoquinovose degradation in Pseudomonas putida SQ1. Proc Natl Acad Sci U S A. 2015;112(31):E4298-305.

2. Frommeyer B, Fiedler AW, Oehler SR, Hanson BT, Loy A, Franchini P, et al. Environmental and Intestinal Phylum Firmicutes Bacteria Metabolize the Plant Sugar Sulfoquinovose via a 6-Deoxy-6-sulfofructose Transaldolase Pathway. iScience. 2020;23(9):101510.

3. Denger K, Weiss M, Felux AK, Schneider A, Mayer C, Spiteller D, et al. Sulphoglycolysis in *Escherichia coli* K-12 closes a gap in the biogeochemical sulphur cycle. Nature. 2014;507(7490):114-7.

4. Liu J, Wei Y, Ma K, An J, Liu X, Liu Y, et al. Mechanistically Diverse Pathways for Sulfoquinovose Degradation in Bacteria. ACS Catalysis. 2021;11(24):14740-50.

5. Liu Y, Wei Y, Zhou Y, Ang EL, Zhao H, Zhang Y. A transaldolase-dependent sulfoglycolysis pathway in Bacillus megaterium DSM 1804. Biochemical and Biophysical Research Communications. 2020;533(4):1109-14.

6. Peck SC, Denger K, Burrichter A, Irwin SM, Balskus EP, Schleheck D. A glycyl radical enzyme enables hydrogen sulfide production by the human intestinal bacterium *Bilophila wadsworthia*. Proc Natl Acad Sci U S A. 2019;116(8):3171-6.

7. Oliveira TF, Vonrhein C, Matias PM, Venceslau SS, Pereira IAC, Archer M. The Crystal Structure of *Desulfovibrio vulgaris* Dissimilatory Sulfite Reductase Bound to DsrC Provides Novel Insights into the Mechanism of Sulfate Respiration*. Journal of Biological Chemistry. 2008;283(49):34141-9.
